# Promising Antiviral Activity of *Agrimonia pilosa* Phytochemicals against Severe Acute Respiratory Syndrome Coronavirus 2 Supported with In Vivo Mice Study

**DOI:** 10.3390/ph14121313

**Published:** 2021-12-16

**Authors:** Nashwah G. M. Attallah, Aya H. El-Kadem, Walaa A. Negm, Engy Elekhnawy, Thanaa A. El-Masry, Elshaymaa I. Elmongy, Najla Altwaijry, Ashwag S. Alanazi, Gadah Abdulaziz Al-Hamoud, Amany E. Ragab

**Affiliations:** 1 Department of Pharmaceutical Sciences, College of Pharmacy, Princess Nourah Bint Abdulrahman University, Riyadh 84428, Saudi Arabia; ngmohamed@pnu.edu.sa (N.G.M.A.); eielmongy@pnu.edu.sa (E.I.E.); naaltwaijry@pnu.edu.sa (N.A.); Asalanzi@pnu.edu.sa (A.S.A.); 2 Egyptian Drug Authority (EDA), Giza 8655, Egypt; 3 Department of Pharmacology and Toxicology, Faculty of Pharmacy, Tanta University, Tanta 31111, Egypt; thanaa.elmasri@pharm.tanta.edu.eg; 4 Pharmacognosy Department, Faculty of Pharmacy, Tanta University, Tanta 31111, Egypt; 5 Pharmaceutical Microbiology Department, Faculty of Pharmacy, Tanta University, Tanta 31111, Egypt; 6 Pharmaceutical Chemistry Department, Faculty of Pharmacy, Helwan University, Helwan 11795, Egypt; 7 Department of Pharmacognosy, College of Pharmacy, King Saud University, Riyadh 11495, Saudi Arabia; galhamoud@ksu.edu.sa

**Keywords:** ALI, immunomodulatory, LPS, LC-ESI-MS/MS, plaque reduction, SARS-CoV-2, TLR-4

## Abstract

The global emergence of the COVID-19 pandemic caused by severe acute respiratory syndrome coronavirus 2 (SARS-CoV-2) has focused the entire world’s attention toward searching for a potential remedy for this disease. Thus, we investigated the antiviral activity of *Agrimonia pilosa* ethanol extract (APEE) against SARS-CoV-2 and it exhibited a potent antiviral activity with IC_50_ of 1.1 ± 0.03 µg/mL. Its mechanism of action was elucidated, and it exhibited a virucidal activity and an inhibition of viral adsorption. Moreover, it presented an immunomodulatory activity as it decreased the upregulation of gene expression of COX-2, iNOS, IL-6, TNF-α, and NF-κB in lipopolysaccharide (LPS)-induced peripheral blood mononuclear cells. A comprehensive analysis of the phytochemical fingerprint of APEE was conducted using LC-ESI-MS/MS technique for the first time. We detected 81 compounds and most of them belong to the flavonoid and coumarin classes. Interestingly, isoflavonoids, procyanidins, and anthocyanins were detected for the first time in *A*. *pilosa*. Moreover, the antioxidant activity was evidenced in DPPH (IC_50_ 62.80 µg/mL) and ABTS (201.49 mg Trolox equivalents (TE)/mg) radical scavenging, FRAP (60.84 mg TE/mg), and ORAC (306.54 mg TE/g) assays. Furthermore, the protective effect of APEE was investigated in Lipopolysaccharides (LPS)-induced acute lung injury (ALI) in mice. Lung W/D ratio, serum IL-6, IL-18, IL-1β, HO-1, Caspase-1, caspase-3, TLR-4 expression, TAC, NO, MPO activity, and histopathological examination of lung tissues were assessed. APEE induced a marked downregulation in all inflammation, oxidative stress, apoptosis markers, and TLR-4 expression. In addition, it alleviated all histopathological abnormalities confirming the beneficial effects of APEE in ALI. Therefore, APEE could be a potential source for therapeutic compounds that could be investigated, in future preclinical and clinical trials, in the treatment of patients with COVID-19.

## 1. Introduction

In December of 2019, a series of individuals in Wuhan, China, developed pneumonia for an unexplained reason [1,2]. The novel coronavirus, the severe acute respiratory syndrome (SARS) corona (COV-2), or COVID-19 was eventually found to be the cause [2,3]. The World Health Organization (WHO) classified this novel coronavirus disease (COVID-19) as an international public health emergency and a difficult public health crisis on 30 January 2020 [4,5]. COVID-19 has been linked to a massive worldwide disease burden [5]. With efforts to prevent and limit its spread, SARS-CoV-2 continues to spread, with deaths exceeding 4.9 million [6]. Modifications in the genetic material of the SARS-CoV-2 virus, which include mutation and recombination, can alter the virus’s life duration, transitivity, cellular tropism, and symptom severity [7]. The discovery of antiviral treatments that target the virus is a key priority because of the serious risk attached.

To lower infectivity, major efforts were made to produce vaccinations and find effective treatments and therapies. There are more than 50 vaccine candidates under development [7,8]. Antiviral extracts and natural components are also being developed to limit virus transmission or prevent infection [9]. Simultaneously, people must know about the benefits of plants in supporting the immune system, as well as to research to build functional foods that can prevent viral infection. This study aimed to evaluate the potential antiviral effects of *Agrimonia pilosa* ethanol extract against the Severe SARS-CoV-2 virus.

From Rosaceae family, *Agrimonia pilosa* Ledeb. is a well-known in traditional Chinese medicine (TCM). It exhibits anti-inflammatory, antiviral, antioxidant, anti-nociceptive, α-glucosidase inhibitory effects, anticancer, and aldose reductase inhibitory activity according to pharmacological research [10,11,12]. The aerial parts of *A. pilosa* were also used as an astringent hemostatic in the Chinese Pharmacopoeia to treat many types of bleeding, including bloody diarrhea, as well as to prevent toxins and reduce swelling in boils and ulcers [13]. A very limited range of *A. pilosa* toxicity tests have been documented regarding their genetic toxicity, acute, and sub-chronic oral toxicity, and systemic safety including the respiratory system, central nervous system, and cardiovascular system [14,15]. Park et al. reported that there are no toxic symptoms upon oral administration of *A. pilosa* were observed and the approximate lethal dose was >2000 mg/kg [15].

Shin et al. reported that *A. pilosa* has broad-spectrum inhibitory activity against three subtypes of human influenza virus [16]. The potential medicinal value, and systemic safety of *A. pilosa*, as well as our continuous interest in the chemistry of biologically active materials from TCM, motivated us to investigate chemical profiling using LC-ESI-MS/MS technique. We also investigated the antiviral activity of *Agrimonia pilosa* ethanol extract (APEE) against SARS-CoV-2 and its immunomodulatory activity. The protective effect of APEE against LPS-induced acute lung injury in mice was also explored.

## 2. Results

### 2.1. Results of LC-ESI-MS/MS Analysis of APEE

A total of 81 compounds were tentatively identified in *A*. *pilosa* alcoholic extract using LC-ESI-MS/MS in positive and negative ion modes. The major compounds belong to flavonoids different classes and coumarins. The complete profile is enlisted in Table 1 and the total ion chromatogram (TIC) of APEE (negative and positive mode) were represented in Supplementary Materials Figure S1.

#### 2.1.1. Characterization of Detected Compounds

**Figure 1:**
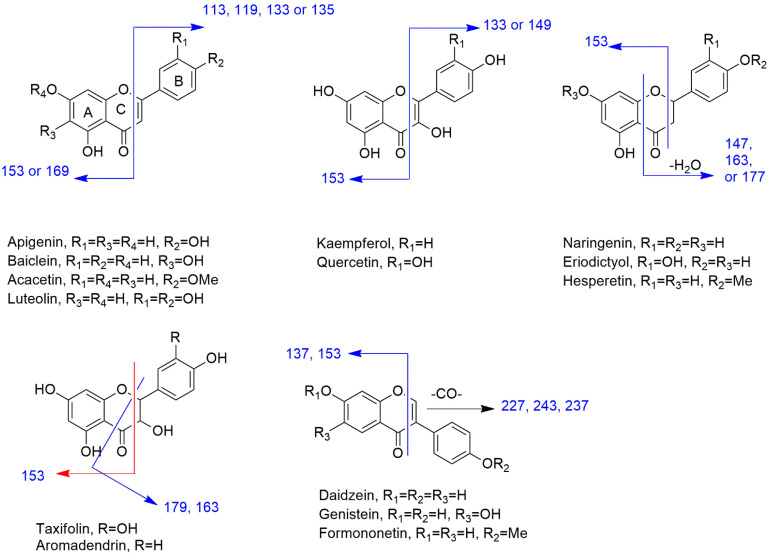
The structures and fragmentation pattern in positive ion mode for the identified aglycones.

LC/MS analysis of APEE revealed that this plant is rich in flavonoid and polyphenolic content. The subclasses flavonols, flavanone, flavone, flavanonol, isoflavonoid, and anthocyanidin were found represented by aglycones and glycosides. The data were found consistent with those in the literature [17,18,19,20,21,22,23]. Figure 1 shows the structures and fragmentation pattern in positive ion mode for the identified aglycones.

##### Flavone Subclass

Luteolin, apigenin, acacetin, and baicalein aglycones and glycosides were confirmed by the corresponding base peak and the fragmentation pattern for each compound. In positive ion mode analysis, the MS/MS fragment at *m*/*z* 153 is characteristic for 5, 7-dihydroxy flavonoids which were detected in luteolin, apigenin, and acacetin. This fragment is increased by 16 D for baicalein due to the hydroxylation at C6. While the MS/MS fragment at *m*/*z* 135 for 3′,4′-dihydroxy flavones is indicative for luteolin. The latter fragment is replaced by the fragment at *m*/*z* 119, 133, and 113 for apigenin, acacetin, and baicalein, respectively due to the fragment ion of ring B with a free hydroxyl group, *O*-methylation or without hydroxylation at C4′ position.

##### Flavonol Subclass

Quercetin and kaempferol aglycones and glycosides are examples of this subclass and are the major flavonoids in *A*. *pilosa*. In positive ion mode analysis, the MS/MS fragment at *m*/*z* 153 confirmed the 5,7-dihydroxy pattern of these aglycones. The MS/MS fragments at *m*/*z* 149 and 133 differentiated quercetin and kaempferol, respectively. The former fragment is characteristic of 3′,4′-dihydroxy flavonol, while the latter fragment is indicative of 3′ or 4′-hydroxyl flavonol.

Rhamnetin, a 7-*O*-methyl quercetin derivative, was identified by the MS/MS fragment ion at *m*/*z* 167 instead of 153 which confirmed ring A *O*-methylation in addition to the fragment ion at *m*/*z* 149. While isorhamnetin was predicted by the MS/MS fragment ions 153 and 163 indicating 5,7-dihydroxy and 3′-*O*-methyl-4′-hydroxy substitutions, respectively.

##### Flavanone Subclass

The flavanone structures in *A*. *pilosa* are naringenin, isosakuranetin, eriodictyol, hesperetin, and their glycosides.

In positive ion mode analysis, the main MS/MS fragment for eriodictyol at *m*/*z* 163 is for 3′,4′-dihydroxy ring B fragment ion after fission of ring C. This fragment is replaced by the fragment ion at *m*/*z* 177 for hesperetin due to 4′-*O*-methylation. In naringenin, this fragment is replaced by the fragment ion at *m*/*z* 147 due to a single hydroxyl group at ring B C4′ position which changed to the fragment at *m*/*z* 161 in isosakuranetin due to 4′-*O*- methylation. The fragment ion at *m*/*z* 153 is the same in the four aglycones due to 5,7-dihydroxylation at ring A.

##### DihydroFlavonol Subclass

Taxifolin and aromadendrin are flavononol compounds detected in *A*. *pilosa*. The 5,7-dihydroxy pattern was confirmed by the MS/MS fragment ion at *m*/*z* 153 in positive ion mode analysis. The MS/MS fragment ion at *m*/*z* 179 is characteristic for dihydroflavonols with 3′,4′-dihydroxy substitution as in taxifolin which changed to *m*/*z* 163 in aromadendrin due to hydroxylation at C4′ only.

##### Flavanols Subclass

Catechin and epicatechin were identified by their [M + H]^+^ ions at *m*/*z* 291 and the fragmentation pattern. Both catechin and epicatechin have similar MS/MS fragmentation in which the fragment ion at *m*/*z* 139 is the most abundant (100%) representing ring A fragment. Ring B fragment yielded a fragment ion at *m*/*z* 123. Catechin and epicatechin have slightly different retention times in LCMS under the conditions used in this study which helped the annotation of both compounds by the database.

##### Isoflavonoids Subclass

Isoflavonoids are characterized by the fragment ion [M + H − CO]^+^ in addition to the fragment ion for ring A. The former ion is at *m*/*z* 227, 243, and 237 for daidzein, genistein, and formononetin, respectively, while the latter ion at *m*/*z* 137 is for daidzein and formononetin and at *m*/*z* 153 for genistein.

Ononin, 7-*O*-glucoside of formononetin, is also detected for the first time in *A. pilosa*. The loss of glucose part yielded the protonated fragment ion at *m*/*z* 269 for formononetin. 

##### Flavonoid Glycosides 

Glycosides were identified through the loss of the sugar yielding the fragment for the respective aglycone. The fragment [M + H − 162]^+^ or [M − H − 162]^−^ indicated the glucosyl unit while the fragment [M + H − 176]^+^ or fragment [M − H − 176]^−^ resulted from the loss of glucouronyl part. Rhamnosyl moiety was recognized by the loss of 146 Da, while arabionosyl and xylosyl motifs were detected by the loss of 132 Da. For C-glucosides, the fragmentation occurs through the breakdown of the C-C bonds within the sugar part. Thus, the fragments [M + H − 120] and [M + H − 90] indicated the C-hexose moiety.

##### Procyanidins

Procyanidin B1 is a dimer of catechin and epicatechin while procyanidin B2 is a dimer of epicatechin. The most common fragmentation of these compounds is the fission of the interflavan linkage yielding the forming units. In this case, the fragment ion at *m*/*z* 291 for catechin and epicatechin is the identifying element of these dimers.

##### Cyanidin Glycosides

The loss of the sugar part resulted in a fragment ion at *m*/*z* 287 which represents an [M^+^] ion of cyanidin.

##### Anthocyanins

Malvidin-3,5-di-*O*-glucoside, malvidin-3-*O*-galactoside, delphinidin-3-*O*-glucoside, and peonidine-3-*O*-glucoside were identified by the loss of 162 Da for hexose units yielding [M^+^] fragment ion at *m*/*z* 331, 303, and 301 for malvidin, delphinidin, and peonidin, respectively.

##### Coumarins

4-Methylumbelliferon and 4-methyl umbelliferyl acetate were detected for the first time in *A*. *pilosa* in addition to esculetin and esculin. 4-Methylumbelliferon yielded an [M + H]^+^ ion at *m*/*z* 177 which gave the fragment ion at *m*/*z* 77. The latter ion is characteristic of a monosubstituted benzoyl structure with an electron negative group. 4-Methyl umbelliferyl acetate lost 43 Da corresponding to the acetyl group to generate an [M^+^] ion for 4-methyl umbelliferon at *m*/*z* 176.

Esculin exhibited a loss of 162 Da for hexose sugar to yield a fragment ion at *m*/*z* 179 for [M + H]^+^ ion of esculetin. Esculetin is characterized by the fragment ion at *m*/*z* 123.

### 2.2. Antioxidant Activity

In the current study, the antioxidant potential of APEE was investigated through radical scavenging, metal-reducing, and oxygen radical absorbance (ORAC) assays. Radical scavenging assays as DPPH and ABTS revealed the activity as (IC_50_ 62.80 µg/mL) and (201.49 mg TE/mg), respectively. In FRAP, a ferric reducing assay, the activity was (60.84 mg TE/mg), and in ORAC (306.54 mg TE/g). The results were displayed in Figures S2–S6.

### 2.3. Total Content of Flavonoids and Polyphenols

The major identified content of total flavonoids was determined as 87.59 mg/g equivalent to rutin while the content of total polyphenols was measured as 237.96 mg/g equivalent to gallic acid. Indicating that APEE is rich in polyphenols and flavonoids.

### 2.4. Antiviral Activity

#### 2.4.1. Cytotoxicity of APEE on Vero-E6 Cells

**Figure 2:**
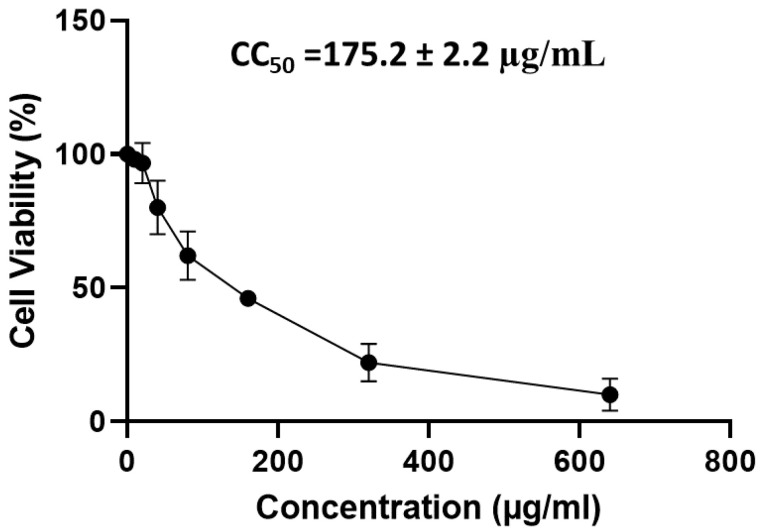
A graph showing the cytotoxicity of APEE on Vero-E6 cells using MTT assay to determine CC_50_. The results are expressed as mean ± SD as the experiments were performed in three independent triplicates.

The CC_50_ of APEE was calculated using MTT assay on Vero-E6 cells to determine the proper APEE concentrations for screening its antiviral activity as shown in Figure 2.

#### 2.4.2. Antiviral Activity of APEE

**Figure 3:**
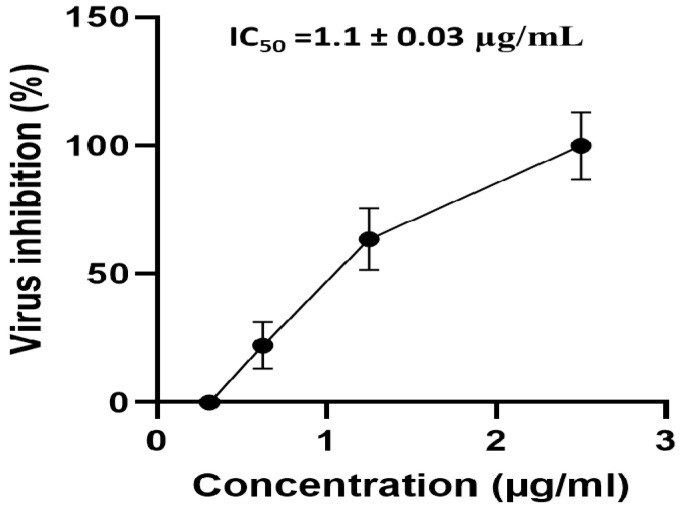
A curve showing the effect of APEE different concentrations on the viability of NRC-03-nhCoV. The results are expressed as mean ± SD as the experiments were performed in three independent triplicates.

APEE showed a promising antiviral activity (90.9% viral inhibition) against the tested SARS-CoV-2 strain (NRC-03-nhCoV) at the highest tested concentration (2.5 µg/mL) and decreased gradually until its antiviral activity against NRC-03-nhCoV was completely diminished at the lowest tested concentration (0.312 µg/mL). The value of IC_50_ against NRC-03-nhCoV is shown in Figure 3.

#### 2.4.3. Mechanism of the Antiviral Activity of APEE against SARS-CoV-2

The percentage of inhibition of different mechanisms of action is presented in Table 2. Remarkably, APEE exerted its antiviral activity against SARS-CoV-2 mainly through a dual mechanism of action; a promising virucidal activity in addition to its interference with the viral adsorption. On the other hand, APEE showed a lower ability to interfere with viral replication.

### 2.5. Immunomodulatory Activity of APEE

#### 2.5.1. MTT Assay on PBMC

**Figure 4:**
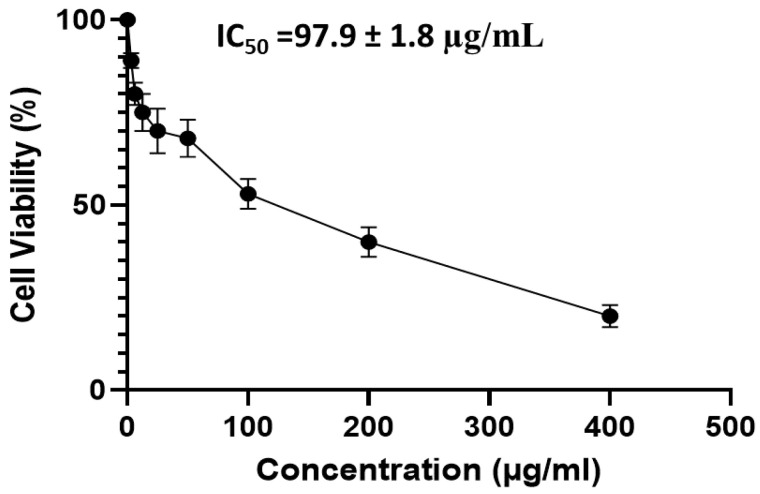
A graph showing cytotoxicity APEE on PBMCs using MTT to determine IC_50_. The results are expressed as mean ± SD as the experiments were performed in three independent triplicates.

The effect of APEE on the viability of PBMC, at concentrations, ranged from 3.125 to 400 μg/mL, was evaluated and its IC_50_ was detected at 97.9 ± 1.8 μg/mL as shown in Figure 4.

#### 2.5.2. qRT-PCR

**Figure 5:**
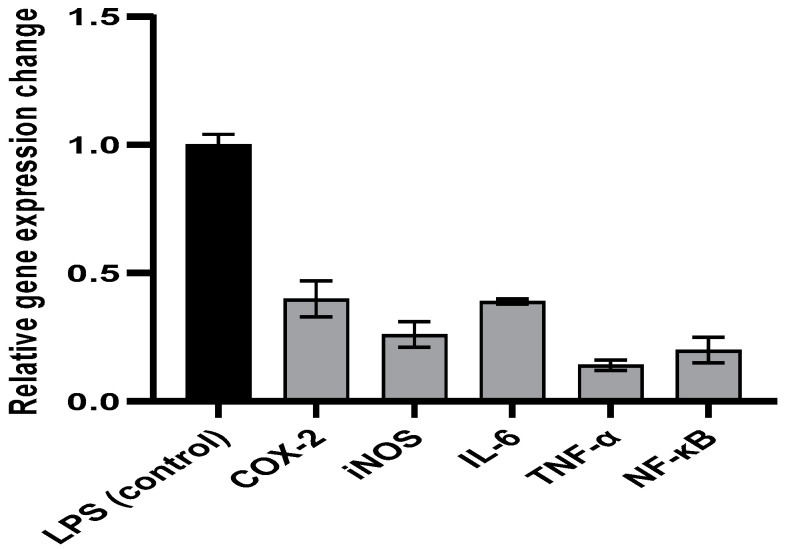
A chart representing the impact of APEE on the expression of the genes encoding COX-2, iNOS, IL-6, TNF-α, and NF-κB in the LPS-induced PBMCs. The results are expressed as mean ± SD as the experiments were performed in three independent triplicates.

The relative expression of the genes encoding enzymes associated with inflammation (COX-2 and iNOS), cytokines related to inflammation (IL-6 and TNF-α), in addition to the transcription factor (NF-κB), was found to be higher in the LPS-induced PBMC. Notably, the APEE treatment of the LPS-induced PBMC, at 0.5 IC_50_, decreased the upregulation of the expression of these genes when compared to non-treated LPS-induced PBMCs as presented in Figure 5.

### 2.6. In Vivo Studies

#### 2.6.1. Effects of APEE Treatment on Lung Wet-Dry Ratio

Table 3 shows that LPS-induced intense pulmonary edema manifested by a remarkable increase in lung W/D ratio (51.13%) compared to normal control. Pre-treatment with APEE 200, 250, and 300 mg/kg induced a significantly lower lung W/D ratio (17.39, 32.17, 26.08%, respectively) in comparison to the LPS group with a more pronounced effect observed in APEE 250 pre-treated group, *p* < 0.05.

#### 2.6.2. Effects of APEE Treatment on MPO Activity

The LPS group showed a marked increase in neutrophil infiltration, confirmed by the elevation in MPO activity (304.27%) compared to normal control. APEE 200, 250, and APEE 300 mg/kg pre-treated groups induced a marked decrease in MPO activity (40, 73.5, and 66.10%, respectively) compared to the LPS group (66.46%) and the effects were superior in APEE 250 group (Table 3), *p* < 0.05.

#### 2.6.3. Effects of APEE Treatment on TAC

LPS treated group induced marked oxidative stress proved by a significant decrease in TAC levels (72.39%) compared to normal control. Preceded treatment with APEE 200, 250, and APEE 300 notably restored TAC levels (69.81, 256.6, and 183%, respectively) compared to the LPS group with a more prominent effect in APEE 250 group (Table 3), *p* < 0.05.

#### 2.6.4. Effects of APEE Treatment on Lung NO Levels

The LPS-induced acute lung injury group showed a pronounced increase in lung NO levels (87.3%) in comparison with the control group. APEE 200, 250, and APEE 300 previously treated groups induced a significant suppression of NO levels (24.57, 44.49, and 36.44%, respectively) compared to the LPS group with a more significant effect in APEE 250 group. (Table 3), *p* < 0.05.

#### 2.6.5. Effects of APEE Treatment on Lung IL-1β Levels

**Figure 6:**
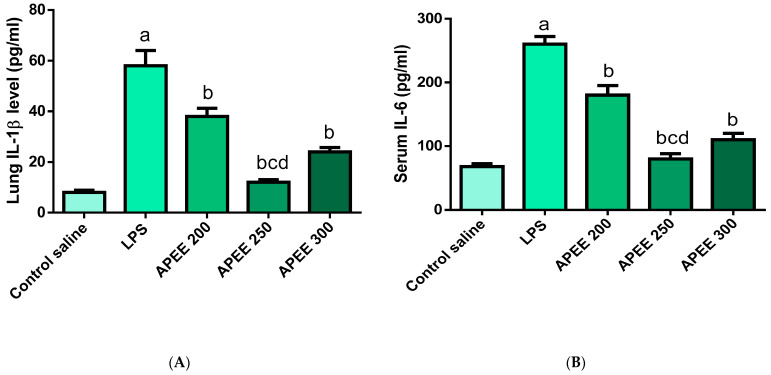

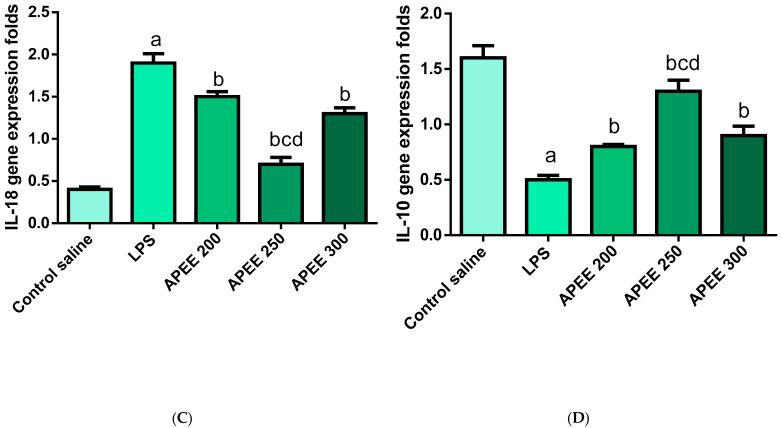
Impact of APEE pre-treatment on (**A**) Lung IL-1β level, (**B**) Serum IL-6 level (**C**) IL-18 gene expression level, (**D**) IL-10 gene expression level. Acute lung injury was urged by I.P. injection of LPS (10 mg/kg). APEE 200, 250, and 300 were given I.P. 30 min before LPS injection. Results were expressed as mean ± SD (*n* = 10/group) as the experiments were performed in three independent triplicates. Significant difference vs. a respective control, b respective LPS group, c respective APEE 200 group, d respective APEE 300 group each at *p* < 0.05.

Figure 6 indicated that the LPS group showed a significant inflammatory response manifested by strong elevation in inflammatory cytokines IL-1β levels (625%) compared to normal control. Previous treatment with APEE 200, 250, and APEE 300 produced significant suppression of IL-1β levels (34.48, 75.86, 58.62%, respectively) compared to the LPS group with a more pronounced effect in APEE 250 group. (Figure 6A), *p* < 0.05.

#### 2.6.6. Effects of APEE Treatment on Serum IL-6 Levels

LPS induced a meaningful elevation in serum IL-6 levels (282%) compared to the control group. Pre-treatment with APEE 200, 250, and APEE 300 significantly brought down IL-6 levels (30, 69.23, and 57.69%, respectively) compared to the LPS group with a more significant effect in APEE 250 group. (Figure 6B), *p* < 0.05.

#### 2.6.7. Effects APEE Treatment on Lung Expression of IL-18 Gene

Figure 6C demonstrated that the LPS group strongly increased IL-18 expression (375%) levels in comparison with the control. APEE 200, 250, and APEE 300 pre-treated groups significantly suppressed IL-18 expression levels (21.05, 63.15, and 31.57%, respectively) compared to the LPS group with a more significant effect in APEE 250 group (Figure 6C), *p* < 0.05.

#### 2.6.8. Effects APEE Treatment on Lung Expression of IL-10 Gene

As shown in Figure 6D, the LPS group significantly suppressed IL-10 expression (68.75%) as opposed to the control group. APEE 200, 250, and 300 pre-treated groups induced a significant upregulation of IL-10 expression levels (37.5, 160, and 44.4%, respectively) compared to the LPS group with a more significant effect in APEE 250 group (160%) (Figure 6D), *p* < 0.05.

#### 2.6.9. Effects APEE Treatment on Lung Expression of HO-1 Gene

**Figure 7:**
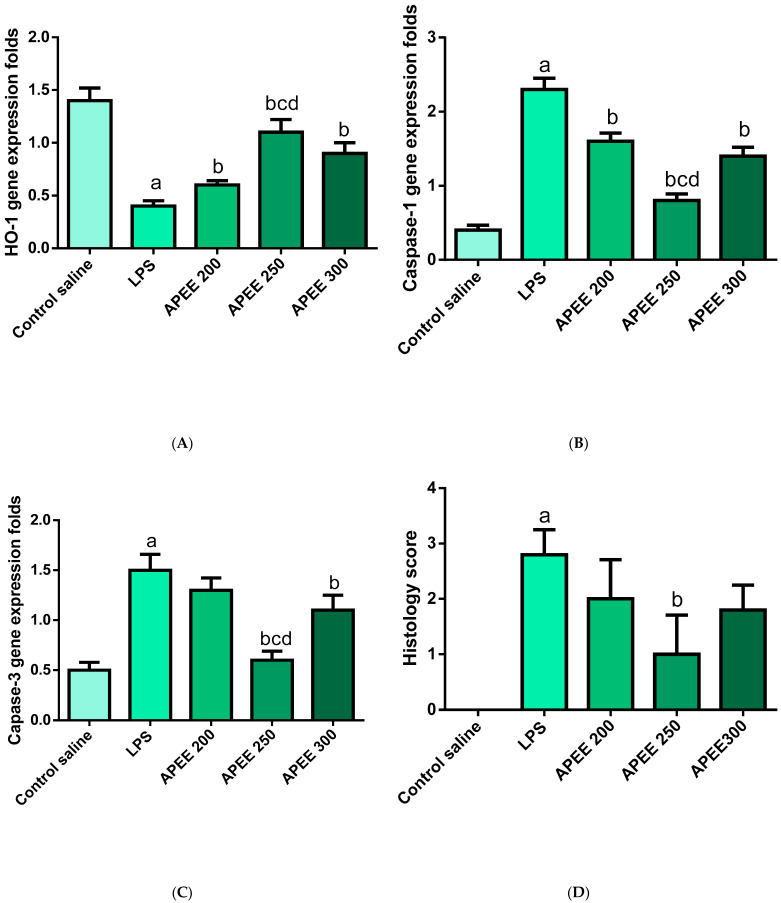
Impact of APEE pre-treatment on (**A**) HO-1 expression level, (**B**) Caspase-1 expression level (**C**) Caspase-3 expression level, (**D**) Lung Histology score. Acute lung injury was urged by I.P. injection of LPS (10 mg/kg). APEE 200, 250, and 300 were given I.P. 30 min before LPS injection. Results were expressed as mean ± SD (*n* = 10/group) as the experiments were performed in three independent triplicates. Significant difference vs. a respective control, b respective LPS group, c respective APEE 200 group, d respective APEE 300 group each at *p* < 0.05.

LPS group induced a noteworthy decrease in HO-1 expression (71.42%) in comparison to the control group. Preceded treatment with APEE 200, 250, and APEE 300 induced a significant elevation of HO-1 expression levels (50, 175, and 125%, respectively) relative to the LPS group and the influence is more prominent in the APEE 250 group (175%) (Figure 7A), *p* < 0.05.

#### 2.6.10. Effects APEE Treatment on Lung Expression of Caspase-1 Gene

LPS treated group significantly increased caspase-1 expression levels (82.6%) relative to the control group. Previous treatment with APEE 200, 250, and APEE 300 produced a distinguishable suppression in caspase-1 expression levels (30.43, 65.21, and 39.13%, respectively) compared to the LPS group with a strong effect in APEE 250 group (Figure 7B), *p* < 0.05.

#### 2.6.11. Effects APEE Treatment on Lung Expression of Caspase 3 Gene

The LPS group produced a notable elevation in lung caspase 3 gene expressions (200%) in comparison with the control. APEE 200, 250, and APEE 300 previously treated groups showed a prominent suppression of caspase-3 expression (13.33, 60, and 26.66%, respectively) compared to the LPS group with a more pronounced effect in APEE 250 group (Figure 7C), *p* < 0.05.

#### 2.6.12. Effect of APEE Treatment on TLR4 Expression

**Figure 8:**
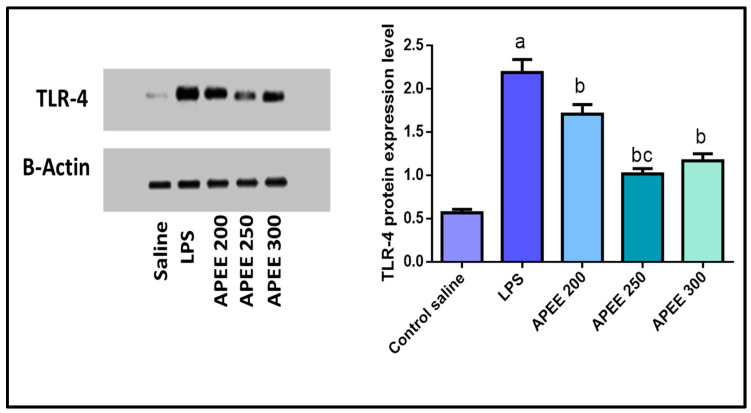
Effect of APEE pre-treatment on the expression of TLR-4 in the lung tissues. The expression levels were measured by western blotting. Acute lung injury was urged by I.P. injection of LPS (10 mg/kg). APEE 200, 250, and 300 were given I.P. 30 min before LPS injection. Results were expressed as mean ± SD (*n* = 10/group) as the experiments were performed in three independent triplicates. Significant difference vs. a respective control, b respective LPS group, c respective APEE 200 group each at *p* < 0.05.

In the current study, TLR4 expression was assessed by Western blot analysis. As shown in Figure 8, LPS significantly up-regulated TLR4 expression (284.21%) compared to the control group. While pretreatment with APEE 200, 250, and 300 significantly decreased the protein expression (21.91, 53.42, and 46.57%, respectively) relative to LPS group. The effect was more significant in APEE 250 group.

#### 2.6.13. Histopathological Examination of the Lung Tissue

**Figure 9:**
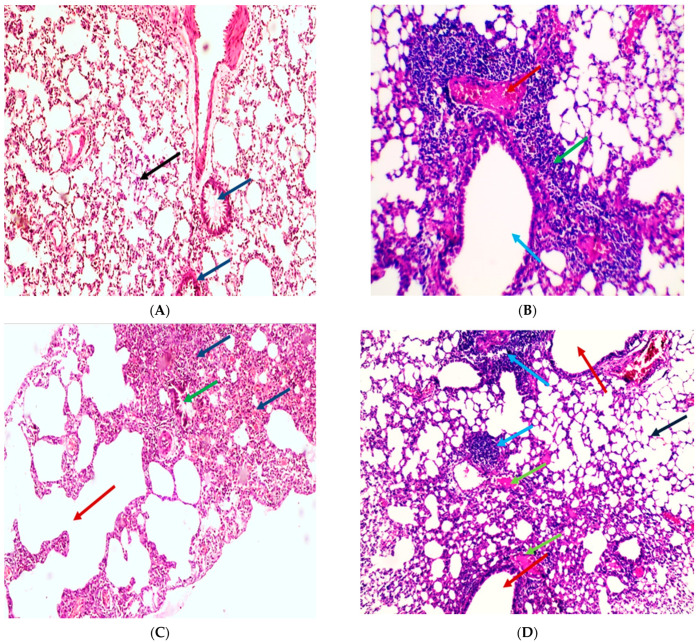

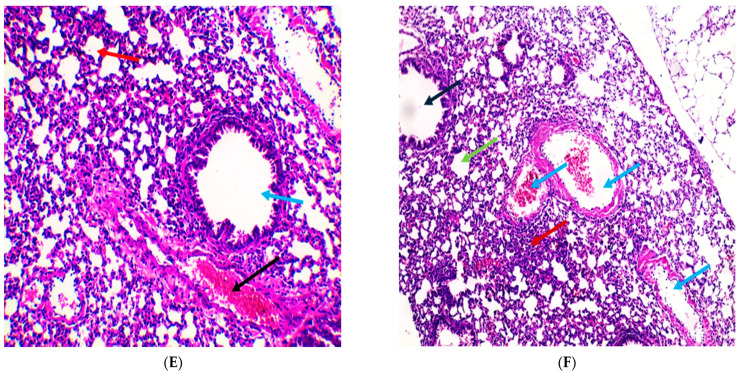
Histopathological examination of H&E-stained sections of lung tissue indicates the influence of APEE treatment on LPS-induced ALI. (**A**) A section in lung of the normal control group indicated normal-sized alveoli separated by fibrous septa (blue arrows) and normal-sized bronchiole (black arrow) (H&E X 100). (**B**) Section in lung of LPS group showed dilated bronchiole (blue arrow) surrounded by marked chronic inflammation and pneumonia (green arrow) and congested vessels (red arrow) (H&E X 200). (**C**) Section in lung of LPS group showed dilated destructed alveolar walls (emphysema) (red arrow) surrounded by destructed bronchioles (green arrow) and alveolar congestion with fibrosis (blue arrows) (H&E X 100). (**D**) Section in lung of APEE 200 treated group showed dilated bronchioles (red arrows) surrounded by decreased interstitial inflammation to moderate degree (blue arrows), congested vessels (green arrows) and decreased emphysema (black arrow) (H&E X 100). (**E**) Section in lung of APEE 250 treated group showed marked remission of inflammation with average-sized of a bronchiole (blue arrow) surrounded by normal-sized alveoli (red arrow) with few congested vessels (black arrow) (H&E X 200). (**F**) Section in lung of APEE 300 treated group showed focal inflammation (red arrow) surrounded by average-sized of a bronchiole (black arrow) surrounded by normal-sized alveoli (green arrow) with many congested vessels (blue arrows) (H&E X 100).

Lung sections in the normal control group showed normal-sized alveoli separated by fibrous septa and normal-sized bronchiole (Figure 9A). While Section in the lung of the positive control group showed dilated bronchiole surrounded by marked chronic inflammation and pneumonia (Figure 9B), dilated destructed alveolar walls (emphysema) surrounded by destructed bronchioles, and alveolar congestion with fibrosis (Figure 9C). In addition, the section in the lung AP 200 treated group showed dilated bronchioles surrounded by decreased interstitial inflammation to a moderate degree, congested vessels, and decreased emphysema (Figure 9D). In addition a section in the lung of the AP 250 treated group showed marked remission of inflammation with average-sized bronchiole surrounded by normal-sized alveoli with few congested vessels (Figure 9E). The section in the lung of the AP 300 treated group showed focal inflammation surrounded by average-sized bronchiole surrounded by normal-sized alveoli with many congested vessels (Figure 9F). In addition, histological lung damage scores were introduced (Figure 7D) and investigated that the most beneficial effects were observed in APEE 250 group.

## 3. Discussion

Medicinal plants have been used for many years as a therapeutic alternative for treating of various disorders such as different viral illnesses [24]. Aqueous and methanol extracts of *A. pilosa* exhibit an antioxidant activity as previously tested [12,25] through the free radical scavenging action. Our investigation is the first report on the activity of *A. pilosa* not only to scavenge free radicals but also to decompose free radicals and to reduce ferric ions. In this study, the IC_50_ in DPPH assay was 62.80 µg/mL, which is about four-to-six-fold of that for methanol and aqueous extracts, respectively [12,25]. This could be attributed to the difference in the total flavonoids and phenolic content which in our study was 87.59 and 237.96 mg/g, mainly due to flavonoid content, while aqueous extract was found to be 243 mg/g and in methanol to be 21.5 mg/g [12,25]. The current results and those previously published revealed that *A. pilosa* possesses a noteworthy antioxidant profile.

In this study, we explored the antiviral activity of APEE on SARS-CoV-2. Pandemic COVID-19 is regarded as a public health threat as there is no existing specific clinically approved antiviral therapy for the management of such disease [26]. It resulted in a large number of deaths globally in addition to serious economic consequences. Several vaccines were promptly developed, approved, and are now being distributed all over the world to control this pandemic disease. However, effective antivirals are still needed for patients who are not vaccinated yet or when the vaccines fail to work efficiently. Treatment of COVID-19, unfortunately, remains largely supportive [27]. Many researchers have conducted studies on the antiviral activity of the currently utilized drugs for different diseases which are called drug repurposing. On the other hand, some researchers investigated the antiviral activity of various plant extracts as many plants are safe for humans and are not expensive [24]. Herein, the antiviral activity of APEE was elucidated in an attempt to participate in the worldwide efforts to find out an effective drug for COVID-19. It was investigated by plaque inhibition assay after the determination of its cytotoxic activity on Vero-E6 cells. These cells are derived from the kidney of an African green monkey and they are widely used in virology studies [28]. APEE exhibited potent antiviral activity against SARS-CoV-2 in Vero-E6 cells with a value of IC_50_ 1.1 ± 0.03 µg/mL. In addition, its mechanism of antiviral activity was investigated. Interestingly, it exhibited a virucidal activity plus its interference with viral adsorption. The inhibition of the viral adsorption is an efficient approach to control infections caused by SARS-CoV-2. Some researchers all over the world have investigated the potential antiviral activity of different plants against SARS-CoV-2 [29,30,31,32]. However, much more investigations on the medicinal plants that could have potential antiviral activity against SARS-CoV-2 are required to face the spreading pandemic COVID-19.

LPS, extracted from Gram-negative bacteria outer membrane, is a strong inducer for PBMC (lymphocytes and macrophages). The LPS-induced macrophages can produce many inflammatory mediators, such as nitric oxide (NO) and prostaglandins. Moreover, these stimulated cells produce various cytokines related to inflammation, such as IL-6 and TNF-α. COX-2 and iNOS enzymes result in the production of prostaglandins and NO by their activity on arachidonic acid and L-arginine, respectively. Thus, the LPS-induced macrophages exhibit an upregulation in the gene expression of COX-2 and iNOS genes [33]. In addition, the NF-κB transcription factor results in an induction of the pro-inflammatory genes for the production of vast amounts of the pro-inflammatory mediators in these activated cells [34]. The overproduction of all these bioactive molecules usually happens in the inflammatory reaction and could lead to damaging outcomes on the tissues. Therefore, the inhibition of such reactions provides a good therapeutic impact to diminish the detrimental effects of inflammation, particularly in the respiratory tract [35]. Hence, we evaluated the immunomodulatory effect of APEE on LPS-induced PBMC. Upregulation of the gene expression of COX-2, iNOS, IL-6, TNF-α, and NF-κB was decreased by treatment of LPS-induced PBMC with APEE in comparison with the non-treated LPS-induced PBMC. An outcome suggests that APEE could be an immunomodulator. The interaction between the host and SARS-CoV-2 has a key role in increasing disease severity as extreme immune response, as a consequence of the viral infection, is frequently accompanied by immune pathogenesis. Thus, APEE could be beneficial in this case.

Acute lung injury is a disease of great importance as it is associated with a high mortality rate [36]. The inflammation plays a pivotal role in ALI pathogenesis. The strongest driving force for the release of inflammatory mediators is LPS [37]. It can activate the host receptor TLR4 resulting in an inflammatory response and ALI [38]. Hence, there is an urgent need to reach an efficacious drug to alleviate lung injury.

*A. pilosa* has been used traditionally for the treatment of abdominal pain, sore throat, headaches, anti-parasitic, and anti-inflammatory agents in Korean medicine. It is also used for wound healing, diminishing wrinkles, and atopic dermatitis. Its root extract is used as a herbal medicine for cancer therapy in Japan. It is routinely applied for the treatment of hepatitis, enteritis, hematochezia, and nephritis caused by bacterial and viral infection [39].

*A. pilosa* is a promising medicinal plant with anti-cancer, antioxidant, and anti-inflammatory effects, and improves glucose tolerance activity [40]. However, the impacts of APEE on LPS-induced ALI and have not yet been examined. For this reason, APEE has been used in this study to explore its protective effects in LPS-induced ALI and investigate the possible anti-inflammatory mechanisms of such protective effects.

The current study revealed that APEE significantly alleviated LPS-induced lung histopathological changes and improved histology scores. In addition, APEE pre-treatment significantly decreased the lung W/D ratio which is used to determine the magnitude of pulmonary edema indicating a substantial inhibition of edema in lung tissue which gave potential for the beneficial effects of APEE in ALI. In the current study, LPS induced marked inflammatory response; it significantly elevated IL-6, IL-1β, and IL-18 levels, and significantly down-regulated IL-10 expression levels. Pre-treatment with APEE significantly suppressed inflammatory cytokine levels and these results were consistent with previous studies [40,41]. Numerous studies gave attention to the anti-inflammatory effects of APEE. In addition, *A. pilosa* had been reported to strongly suppress NO production, PGE2 expression levels, and the release of inflammatory mediators IL-1β and IL-6, in RAW264.7 cells [39,42]. The underlying mechanisms of the anti-inflammatory effect of APEE may be due to their flavonoids which were proven previously to have strong anti-inflammatory effects. According to data obtained from LC/MS, it is concluded that APEE contains numerous flavonoids including but are not limited to quercetin, isoquercitrin, hesperetin, luteolin, rutin, kaempferol, apigenin, and esculin and all have strong anti-inflammatory and antioxidant effects [40,43,44] which may explain the promising protective effects of APEE in LPS-induced ALI due to its enormous components of valuable flavonoids.

Toll-like receptor-4 (TLR-4), a pattern recognition receptor, could recognize LPS from Gram-negative bacteria. It activated IL-1β release and upregulated IL-1RI expression through NF-κB-dependent signaling [37]. This work investigated that LPS-induced marked elevation in TLR-4 expression and these high levels were greatly down-regulated with APEE treatment and these results agree with other results [41].

In the early phase of ALI, neutrophils are recruited at the site of injury-inducing cytotoxic effects are assessed by MPO activity. In the current study, LPS induced a remarkable increase in MPO activity, treatment with APEE significantly reduced MPO activity, and these effects are in line with other reports [40].

Several reports have confirmed the role of oxidative stress in all stages of COVID-19 pathogenesis, cytokine storm cycle, blood clotting mechanism, and aggravating hypoxia [45,46,47]. Taken together, this evidence confirmed the important participation of oxidative stress in the pathogenesis of viral infection in all stages. In addition, they reported crosstalk between oxidative stress and the cytokine storm as a mechanism that sustains and worsens the tissue injury, which is terminated by hypoxia and organ failure. Since the importance of oxidative stress on all stages of COVID-19 pathophysiology was confirmed, it was very important in our study to confirm the antioxidant potential of APEE in-vivo to support the finding of our study.

In our study LPS-induced pronounced oxidative stress which is evidenced by the downregulation of HO-1 expression levels, while treatment with APEE significantly restored normal HO-1 expression levels and mitigated oxidative stress conditions induced by LPS. In addition, LPS-induced oxidative stress was confirmed by reducing TAC and NO elevation in lung tissues. APEE treated group restored TAC levels and decreased NO levels and these antioxidant effects are in accordance with other studies [40,43].

The underlying mechanisms of these promising antioxidant effects of APEE may be attributed to their numerous flavonoids as reported by LC/MS which were reported previously with their powerful antioxidant properties [41,46]. Apoptosis of lung endothelial and epithelial cells is a critical event in the development of ALI, and this was confirmed by the protective role of caspase inhibitor against ALI in mice [48].

According to our results, LPS induced prominent up-regulation of caspase-3 gene expression and these values were strongly down-regulated in APEE treated groups which were confirmed in other studies [49]. Regarding the importance of apoptosis in ALI and its role in mitigating inflammation associated with lung injury via increasing neutrophil apoptosis. As a result, apoptosis provides a way to remove neutrophils from the inflammation area inducing minimal damage to the surrounding tissue [48]. Based on this information, the anti-apoptotic effect of APEE may explain at least in part their anti-inflammatory effects. Caspase-1 has a dual role in apoptotic processes and is a key factor in the generation and maturation of pro-inflammatory cytokine IL-1β and IL-18 from their precursor [37].

According to the results of this study, PS induced a significant increase in caspase -1 expression comparing to the control group. In addition, APEE treated groups showed a marked reduction in caspase-1 expression in lung tissues. The inhibitory effect of APEE on caspase-1 expression may explain its anti-inflammatory effects in ALI by decreasing pro-inflammatory cytokines maturation and release.

A previous study [50] investigated the antiviral activity of *A. pilosa* and *Galla rhois*, in addition to their mixture on SARS-CoV-2 using plaque reduction assay. They also oanalyzed the supernatants of the virus-infected cells for the spike proteins of SARS-CoV-2. In addition, they performed a molecular docking simulation to predict the binding between the SARS-CoV-2 spike receptor-binding domain (RBD) and the active constituents of the mixture. In our study, we investigated the multi-effects of APEE to explore its effectiveness in the treatment of COVID-19 patients who suffer from the virus in addition to the destruction of the lung. Thus, we elucidated the antiviral activity of APEE in addition to its antiviral mechanisms of action. Moreover, its immunomodulatory activity in LPS-induced PBMC and its lung protective effect in LPS-induced acute lung injury in mice were assessed.

## 4. Materials and Methods

### 4.1. Plant Materials and Extract Preparation

*Agrimonia pilosa* Ledeb. aerial parts were purchased from Bozhou Swanf Commercial and Trade Co., Ltd., Anhui, China. The identification of the plant was confirmed by Esraa Ammar, Plant Ecology lecturer, Botany Department, Faculty of Science, Tanta University. A voucher specimen (PGA-AP-122-W) was kept in the herbarium of the Department of Pharmacognosy, Tanta Pharmacy.

The plant powdered material (950 g) was extracted with ethanol using cold maceration (three times, 7 liters of ethanol). The extract was concentrated under reduced pressure to afford a residue (18.46 g), the extract was kept in the refrigerator for further biological investigations.

### 4.2. Animals

A total of 50 adult male Swiss albino mice (22–25 gm) were utilized in the current study (animal house College of Veterinary Medicine, Cairo). All mice were hosted in normal cages with easy access to standard pellet and filtered water under controlled temperature conditions (25 ± 2 °C) and illumination (12-h light/dark cycle). Mice were acclimatized for 1 week before starting the experiment. All procedures were carried out in accordance with the Research Ethical Committee and experimental protocols in conformity with the rules for the care and use of laboratory animals (Faculty of Pharmacy, Tanta University, Egypt, Approval NO.PO 00102).

### 4.3. Drugs and Chemicals

This study utilized Lipopolysaccharides (Sigma, MO, USA). 2,2-Diphenyl-1-picrylhydrazyl (DPPH), 2,2′-azinobis (3-ethylbenzothiazoline-6-sulfonic acid) diammonium salt (ABTS), 6-hydroxy 2,5,7,8-tetramethylchroman-2-carboxylic acid (Trolox), 2,2′-azobis (2-methylpropionamidine) dihydrochloride (AAPH), phosphate buffer, 2,4,6-tri(2-pyridyl)-s-triazine (TPTZ), iron (III) chloride hexahydrate, and Folin–Ciocalteu reagent were bought from Sigma–Aldrich. All used chemicals and solvents were purchased from Sigma–Aldrich and were of high analytical grade (St. Louis, MO, USA).

### 4.4. LC-ESI-MS/MS Analysis of APEE

Compounds in the APEE were detected by Proteomics and Metabolomics Unit, Children’s Cancer Hospital (57357), Basic Research Department, Cairo, Egypt. Adopting the criteria described by Attallah et al. [51]. For HPLC separation, A (Waters) reversed-phase X select HSS T3 column (diameters are 2.1 × 150 mm, 2.5 μm), a (Phenomenex) precolumn, and in-Line filter discs (0.5 μm × 3.0 mm) were employed. To identify compounds, PeakView^TM^ software was used to compare retention duration and *m*/*z* values obtained by MS and MS^2^. The XIC Manager in PeakView^TM^ software was used to calculate peak area values. Extracted ion chromatograms (XICs) for each targeted analyte were automatically created and compared to a user-defined threshold [51].

### 4.5. Antioxidant Activity of APEE

#### 4.5.1. Determination of Total Flavonoids and Polyphenolics Content

The total flavonoid content was determined by colorimetric analysis of serial dilutions of the extract using the aluminum chloride methodology and rutin as a standard. With gallic acid as a reference, the total amount of polyphenols was determined using the Folin–Ciocalteu procedure. For each method, the measured contents were reported as mg/g equivalent of the respective standard [52,53].

#### 4.5.2. The DPPH Radical Scavenging Capacity

The DPPH radical scavenging capacity of APEE was evaluated according to the method of Boly et al. [54]. In a 96 well plate (*n* = 6), 100 µL of freshly made DPPH reagent (0.1% in MeOH) was added to 100 µL of the sample, and the reaction was incubated at room temperature for 30 min in the dark. The consequent decrease in DPPH color intensity was measured at 540 nm at the ending of the incubation time. A stock solution of 100 µM of Trolox was prepared in MeOH from which seven concentrations were prepared including 50, 40, 30, 20, 15, 10, and 5 µM. The following equation is used to represent data as means SD:

$$\begin{array}{cc} percentage & \;inhibition\\ & =\frac{\left(\mathrm{Average}\;\mathrm{absorbance}\;\mathrm{of}\;\mathrm{blank}-\mathrm{average}\;\mathrm{absorbance}\;\mathrm{of}\;\mathrm{the}\;\mathrm{test}\right)}{\mathrm{Average}\;\mathrm{absorbance}\;\mathrm{of}\;\mathrm{blank}}\times 100 \end{array}$$

Microplate reader FluoStar Omega was used to recording the results. The IC_50_ value was calculated using Graph pad Prism 5^®^ by converting the concentrations to logarithmic values and selecting the non-linear inhibitor regression equation (log (inhibitor) versus normalized response—variable slope equation) [55]. (Figure S2)

#### 4.5.3. The ABTS Radical Scavenging Capacity

The assay was carried out according to the method of Arnao et al. [56]. Eight serial dilutions of Trolox were prepared in the concentrations of 700, 600, 500, 400, 300, 200, 100, and 50 µM, while the sample was prepared at a concentration of 0.5 mg/mL in MeOH. The results are displayed as µM Trolox equivalents (TE)/mg samples using the linear regression equation extracted from the calibration curve (linear dose–inhibition curve of Trolox) (Figure S3).

#### 4.5.4. Ferric Reducing Antioxidant Potential (FRAP) Assay

Ferric reducing ability assay was performed according to Benzi et al. [57]. A freshly made TPTZ reagent (300 mM acetate buffer (pH = 3.6), 10 mM TPTZ in 40 mM HCl, and 20 mMFeCl_3_, respectively, in a ratio of 10:1:1 *v*/*v*/*v*). In a 96-well plate (*n* = 3), 190 µL of newly made TPTZ reagent was mixed with 10 uL of the sample, and the reaction was incubated in the dark at room temp. for 30 min. A generated blue color was detected at 593 nm at the end of the incubation time. The ferric reducing ability of the samples result is displayed as µM TE/mg sample using the linear regression equation extracted from the following calibration curve (linear dose–response curve of Trolox). Means and standard deviations are used to represent the data (Figure S4).

#### 4.5.5. Free Radical Scavenging by the Oxygen Radical Absorbance Capacity (ORAC) Assay

We followed the procedure of Liang et al. [58] with some changes. Briefly, 12.5 µL of the produced sample(s) were incubated with 75 µL fluoresceine (10 nM) for 30 min at 37 °C. For background measurements, three cycles (485 EX, 520 EM, nm) of fluorescence measurement (485 EX, 520 EM, nm) were performed (cycle time, 90 sec). After then, each well received 12.5 µL of newly produced 2,2′-Azobis(2-amidinopropane) dihydrochloride (AAPH) (240 mM). The measurement of fluorescence (485 EX, 520 EM, nm) was conducted for 2.5 h (85 cycles, every 90 s). The results are presented as µM TE/mg sample using the linear regression equation extracted from the following calibration curve (linear dose–inhibition curve of Trolox) (Figure S5).

### 4.6. Antiviral Activity of APEE

#### 4.6.1. Virus and Cell Lines

Vero-E6 cells (Vacsera, Cairo, Egypt) were utilized in the current study to propagate SARS-COV-2, hCoV-19/Egypt/NRC-03/2020 (Accession Number on GSAID: EPI_ISL_430820) virus “NRC-03-nhCoV”. The cells were cultured in Dulbecco’s modified Eagle’s medium (DMEM) supplied with 10% fetal bovine serum (FBS) (Merck) plus1% penicillin/streptomycin mixture (Merck). They were incubated at 37 °C in the presence of 5% CO_2_. For the generation of the virus stock, cells were spread into tissue culture flasks 24 h before the infection with hCoV-19/Egypt/NRC-3/2020 isolate at a multiplicity of infection of 0.1 in the infection medium which was composed of DMEM containing 2% FBS, 1% penicillin/streptomycin, in addition to 1% trypsin treated with L-1-tosylamido-2-phenylethyl chloromethyl ketone. After 2 h, the infection medium with the viral inoculum was withdrawn and a fresh infection medium was added and incubated for 3 days. After that, the cell supernatant was centrifuged at 2500 rpm for 5 min for removing the cell debris and it was transferred to a falcon tube, and aliquoted, then titrated using plaque infectivity assay.

#### 4.6.2. MTT Cytotoxicity Test

Stock solutions of APEE, in ddH_2_O, were used to determine its half-maximal cytotoxic concentration (CC_50_). Working solutions of APEE were produced via diluting the APEE stock solutions using DMEM (high glucose). Vero-E6 cells were used to determine the cytotoxic activity of APEE using the 3-(4,5-dimethylthiazol-2-yl)-2,5-diphenyltetrazolium bromide (MTT) method as previously described [26]. The absorbance of the formed formazan was measured at an optical density (OD) of 540 nm using ELISA reader (Sunrise Tecan, Männedorf, Switzerland). The cytotoxicity percentage was calculated according to the subsequent formula:

$$\%\;\mathrm{cytotoxicity}=\frac{\left(\mathrm{the}\;\mathrm{absorbance}\;\mathrm{of}\;\mathrm{cells}\;\mathrm{without}\;\mathrm{treatment}-\mathrm{absorbance}\;\mathrm{of}\;\mathrm{cells}\;\mathrm{with}\;\mathrm{treatment}\right)\times 100}{\mathrm{the}\;\mathrm{absorbance}\;\mathrm{of}\;\mathrm{cells}\;\mathrm{without}\;\mathrm{treatment}\;}$$

A graph was constructed between % cytotoxicity and sample concentration for calculation of the concentration that resulted in 50% cytotoxicity (TC_50_).

#### 4.6.3. Plaque Inhibition Assay

Plaque inhibition assay for testing the antiviral activity of APEE was performed according to the method previously described [59]. It was conducted in a six-well plate with 90% confluent Vero-E6 cells. In brief, after 10-fold serial dilution of the propagated virus using DMEM without any additives, 100 µL of each dilution was blended with DMEM (400 µL) to be added to Vero-E6 cells and incubated at 37 °C, for only one hour, in the presence of 5% CO_2_ to permit viral adsorption. After that, the virus inoculum was withdrawn and Vero-E6 cells were overlaid with 3 mL of DMEM containing 2% agarose and APEE. The plates were left for 10 min to permit the agarose to solidify and then incubated at 37 °C in the presence of 5% CO_2_ for 3 days. Formalin (10%) was used as a fixer, and it was added to each well of the plate for one hour. After discarding the fixer, the wells were washed with water left to dry. Finally, a crystal violet stain (0.1%) was utilized to stain each well, then they were rinsed with water and left to dry. The plaques were clear unstained spots in a violet cellular background. Control wells containing the untreated virus with Vero-E6 cells were included. At last, counting of the plaques was performed to calculate the percentage of reduction in plaque formation according to the subsequent formula:

% inhibition = viral count (untreated) − viral count (treated)/viral count (untreated) × 100

#### 4.6.4. Mechanism(s) of Action

The following protocols were carried out to investigate the main mechanism of action of APEE against NRC-03-nhCoV.

##### Viral Adsorption

The mechanism of viral adsorption was tested as previously described [60] with minor modifications. After overnight cultivation of Vero-E6 cells (10^5^ cells/mL) in a 6-well plate at 37 °C, APEE was added to the cells in 200 µL DMEM without any additives and left for 2 h in the refrigerator (at 4 °C). Then, the non-absorbed APEE was discarded, and the cells were washed three times using DMEM without any additives. NRC-03-nhCoV virus was diluted to 10^4^ PFU/well and was added to the pretreated cells for one hour, then they were overlaid with 3 mL of DMEM containing 2% agarose. The plates were allowed to solidify, and they were incubated at 37 °C till the formation of the viral plaques. Finally, the formed plaques were fixed using formalin solution (10%) for one hour and stained using crystal violet. The percentage of reduction in plaque formation was calculated as previously mentioned.

##### Viral Replication

APEE effect on the viral replication was detected as previously described [61]. In brief, after overnight cultivation of Vero-E6 cells (10^5^ cell/mL) in a 6-well plate at 37 °C, NRC-03-nhCoV was added to the cells and incubated for one hour at 37 °C. The cells were carefully rinsed three times using DMEM without any additives for eliminating the non-adsorbed viral particles. Then, APEE was added in different concentrations to the infected cells, and they were incubated for another hour. The inocula containing APEE was then removed and 3 mL DMEM containing 2% agarose was added to the cells and they were incubated at 37 °C to allow solidification and formation of the viral plaques. The formed plaques were then fixed and stained as previously mentioned and the percentage of inhibition of plaque formation was calculated in comparison with the control wells as stated formerly.

##### Virucidal Activity

This mechanism was assayed according to the previously described protocol [62]. After overnight cultivation of Vero-E6 cells (10^5^ cells/mL) in a six-well plate at 37 °C, 200 µL of DMEM, without supplements, containing NRC-03-nhCoV was added and incubated for one hour. Then, this mixture was 10-fold diluted three times using DMEM without supplements. One hundred microliters of each one of the prepared dilutions were added to the Vero-E6 cells and left for one hour. DMEM including 2% agarose was used to overlay Vero-E6 cells and incubated at 37 °C for solidification and formation of the viral plaques. The formed plaques were then fixed and stained as previously mentioned and the percentage of inhibition in plaque formation was calculated in comparison with the control wells as stated formerly.

### 4.7. Immunomodulatory Activity

#### 4.7.1. Peripheral Blood Mononuclear Cells (PBMCs) Isolation

They were isolated from healthy donor’s blood via ficoll density gradient centrifugation and the PBMCs were cultured in six-well plates using Roswell Park Memorial Institute (RPMI 1640) medium with 10% heat-inactivated FBS, 2 mM L-glutamine, and 1% penicillin-streptomycin solution. Then, they were incubated overnight at 37 °C in an atmosphere of 5% CO_2_ for maintenance of the cells.

#### 4.7.2. MTT Cytotoxicity Assay

The toxicity of APEE on PBMC, at concentrations, ranging from 3.125–100 µg/mL, was evaluated using the MTT test as previously described after incubating the PBMC (at a concentration of 5 × 10^4^ cells/mL) with APEE for 24 h [63]. APEE mean inhibitory concentration (IC_50_) on PBMC was detected and the immunomodulatory effect of APEE was assessed in the (LPS)-activated PBMC at 1/2 IC_50_. PBMC treated with 100 ng/mL LPS alone or with 0.05% DMSO were used as controls.

#### 4.7.3. Quantitative Real-Time PCR (qRT-PCR)

The impact of APEE on the relative gene expression of cyclooxygenase-2 (COX-2), nitric oxide synthase (iNOS), interleukin-6 (IL-6), tumor necrosis factor-alpha (TNF-α), and nuclear factor kappa B (NF-κB) in LPS-stimulated PBMC was investigated [64]. In brief, 2 × 10^6^ cells/mL were grown in RPMI 1640 medium in six-well plates and after overnight incubation, PBMCs (at a concentration of 1 × 10^6^ cells/mL) were treated with 100 μL of LPS (20 ng/mL) extracted from *Escherichia coli* O127:B8 for 24 h in the presence and absence of 0.5 IC_50_ of APEE. The impact of APEE on the gene expressions of COX-2, iNOS, IL-6, TNF-α, and NF-κB was assessed by qRT-PCR (primers are shown in Table S1). The fold change in gene expression was estimated by the 2^−ΔΔCT^ method [64].

### 4.8. Induction of Acute Lung Injury by LPS

Acute lung injury was induced by LPS as described previously [37]. After one week of acclimatization, all mice were arbitrary allocated to five groups (*n* = 10 per group), and the drugs were injected intraperitoneally (I.P.): Group I: control group (30 min saline 0.9% + saline 0.9%), Group II: LPS group [mice were given saline 30 min before LPS injection (10 mg/kg)], Group III: LPS + APEE group (mice were given APEE 200 mg/kg 30 min before LPS treatment (10 mg/kg)), Group IV: LPS + APEE group (mice were given APEE 250 mg/kg 30 min before LPS injection (10 mg/kg)), and Group V: LPS + APEE group [mice were given APEE 300 mg/kg 30 min before LPS treatment (10 mg/kg)]. The doses used in this study were according to the data from previous reports. A study by Nho et al. reported the use of *A. pilosa* in doses 100 and 500 mg/kg [65]. In addition, Park et al. reported the use of *A. pilosa* in dose 200 mg/kg as anti-nociceptive [66]. In our study, three dose levels were used to determine the most protective dose that safeguards against LPS-induced ALI in mice.

#### 4.8.1. Sample Collection

Two hours following LPS treatment, the mice were anesthetized by diethyl ether then blood was collected via cardiac puncture into a syringe and then, was centrifuged at 3000 rpm for 10 min. Serum was delicately removed and kept at −20 °C till utilized for measurement of total antioxidant enzyme capacity (TAC) and IL-6. Then, mice were killed by cervical dislocation and lung tissues were collected. One part was used for histopathological examination and the second part was used for the measurement of biochemical parameters.

#### 4.8.2. Measurement of Lung Wet/Dry Ratio

The degree of lung edema was evaluated by the wet/dry ratio (W/D ratio). The wet weight of the lungs was assessed, following that the lungs were dried in an oven (60 °C for 72 h) to maintain a dry weight. Eventually, the W/D ratio was estimated using the wet and dry weights [37].

#### 4.8.3. Determination of Total Antioxidant Enzyme Capacity (TAC)

The TAC was determined calorimetrically in serum samples according to the manufacturer protocol (TAC kit, Bio diagnostic company, Egypt). In brief, 200 µL of the serum was blended with 500 µL of H_2_O_2_. Then mixed and incubated for 5 min at 37 °C [67]. The TAC was proportional to the intensity of the colored product which is measured at 505 nm using double beam spectrophotometer (Shimadzu UV-PC 1601, Kyoto, Japan)

#### 4.8.4. Determination of Lung Nitric Oxide (NO) Content

The NO content was measured in lung tissue homogenate according to the method reported earlier (2). Absorbance was measured at 540 nm using a double beam spectrophotometer (Shimadzu UV-PC 1601, Kyoto, Japan). Total NO concentration in each sample was assessed by the sodium nitrite standard curve. 

#### 4.8.5. Determination of Lung Myeloperoxidase Activity (MPO)

As a neutrophil infiltration marker, MPO activity was assessed as described earlier [68]. The principle of such a method is the kinetic evaluation of the yellowish-orange color of the oxidation product of o dianisidine with MPO in the vicinity of H_2_O_2_. The change in absorbance was reported at 460 nm using a double beam spectrophotometer (Shimadzu UV-PC 1601, Kyoto, Japan)

#### 4.8.6. Determination of Lung IL-1β Levels

The level of inflammatory mediators IL-1β in lung tissues was assessed according to the manufacturer protocol. The level of IL-1β was assessed according to the method described in commercial ELISA kits (Abcam Co., Waltham, MA, USA).

#### 4.8.7. Determination of Serum IL-6 Levels

Serum IL-6 was assessed according to the manufacturer’s instructions. The values of IL-6 were determined according to the method described in commercial ELISA kits (Abcam Co., Waltham, MA, USA).

#### 4.8.8. qRT-PCR for IL-10, IL-18, HO-1, Capase-1, and Caspase-3 Genes

TRIzol solution (Thermo Fisher, Waltham, MA, USA) was utilized to isolate total RNA from lung samples according to the manufacturer’s instructions. PrimeScript™ RT Reagent kit (Takara Bio Inc, Kusatsu, Shiga, Japan) was applied to construct cDNA at 42 °C for 15 min and 85 °C for 5 s. Then, qPCR was performed using SYBR^®^ Green qPCR assay (Thermo Fisher Scientific, Waltham, Massachusetts, USA). Table S2. showed used primer probes under study. By using ABI 7000 RT PCR machines qPCR was performed with the following qPCR conditions: Initial denaturation at 95 °C for a 3 min, followed by 40 PCR cycles (95 °C for 5 s, 60 °C for 20 sec and 72 °C for 20 s). Finally, the 2^−ΔΔCT^ method was performed to measure relative mRNA expression and normalized to β-actin [69].

#### 4.8.9. Western Blot Analysis for Toll-Like Receptor-4

Samples total soluble proteins were fragmented on 10% SDS polyacrylamide gels and transferred to a Hybond^™^ nylon membrane (GE Healthcare) via TE62 Standard Transfer Tank with Cooling Chamber (Hoefer Inc. and incubate for one hour at room temperature. Then, 5% skim milk (BD, Franklin Lakes, NJ, USA) was added as blocking solution for 60 min and incubated with primary antibodies against TLR-4 primary antibody (1:500, Abcam, MA, USA) and β-actin (1:1000, Abcam, MA, USA) overnight at 4 °C. Phosphate buffered saline solution with a low concentration detergent solution such as 0.05% to 0.1% tween 20 (PBST) was used for triplicate washes then incubated for one-hour HRP-conjugated secondary antibody (Antibody concentration 0.1–0.5 μg/mL). Antibody concentration was adjusted from 0.05 to 2.0 μg/mL to obtain the desired signal strength and low background. Finally, the signal was detected using ECL western blotting substrate (Proteinsimple, FluorChem E) [70,71].

#### 4.8.10. Histopathological Examination of Lung Sections

At the end of the study, the lung was excised, washed with phosphate-buffered saline, and used for histopathological examination. Lung sections were fixed in 10% formalin solution (pH 7.4) for 24 h and then processed in ascending grades of alcohol then xylene. The tissues were eventually fixed for 24 h in paraffin wax at 65 °C. Tissue blocks were sectioned at 4 μm thickness and then stained by hematoxylin and eosin (H&E) and then inspected by a light microscope. The histology scoring assessed parameters involved edema, intra-alveolar cell infiltration, alveolar congestion, and hemorrhage. The scoring of each item was recorded as the following grades: normal (0), mild (1), moderate (2); and severe (3) [37].

#### 4.8.11. Statistical Analysis

Data were represented as mean ± SD. Regression analysis was performed, and correlation coefficients were defined, for all standard curves. Comparisons between different groups were conducted by one-way analysis of variance (ANOVA) followed by a Tukey–Kramer post-hoc test. The level of significance was set at *p* < 0.05. The statistical analyses were carried out by Prism version 9 (GraphPad Software, Inc, San Diego, CA, USA).

## 5. Conclusions

Owing to the continued global expansion of COVID-19 that is caused by SARS-CoV-2, effective antiviral drugs are highly needed to treat patients especially those with a high risk of life-threatening disease. In the current study, we demonstrated in vitro evidence for the potent antiviral activity of APEE against SARS-CoV-2 with IC_50_ 1.1 ± 0.03 µg/mL. The mechanism of antiviral activity of APEE was investigated and it was found to inhibit the viral absorption and have virucidal activity. In addition, APEE exhibited a promising immunomodulatory activity on LPS-induced PBMC by attenuation of the gene expression upregulation of COX-2, iNOS, IL-6, NF-κB, and TNF-α.

This present study also demonstrated that APEE showed a promising protective effect against LPS-induced ALI by its anti-inflammatory, antioxidant, and anti-apoptotic effects and the underlying mechanisms for these valuable effects may be attributed to diminishing TLR-4 expression, Caspase-1/IL-1β activation, inflammatory cytokines, and restoring IL-10 and HO-1 expression levels.

However, in silico studies could be beneficial to demonstrate the binding affinity of APEE to the targets of SARS-CoV-2. Future preclinical and clinical investigations should be performed on APEE to demonstrate its efficacy in the treatment of the current pandemic COVID-19. In addition, APEE could be combined with various antiviral drugs and assessed for their antiviral activity.

## Figures and Tables

**Table 1:** Phytochemical profiling of *A*. *pilosa* by LC-ESI-MS/MS in negative and positive mode.

No.	R_t_ min	[M − H]^−^	[M + H]^+^	MS^2^ Ions *m*/*z*	Identification
1	1.14	173		83, 155, 173	Shikimic acid
2	1.43		179	123, 179	Esculetin
3	2.25	359		117, 134, 163, 176, 181, 185, 290, 359	Rosmarinic acid
4	4.06		229	137, 229	Resveratrol
5	4.52		579	84, 123, 127, 135, 139,147, 161, 163, 229, 243, 253, 257, 271, 273, 275, 283, 287, 289, 291, 299, 301, 391, 409, 421, 589	Procyanidin B1
6	4.77		291	115, 119, 123, 139, 143,147, 161, 165, 207, 291	Catechin
7	5.16		611	267, 287, 303, 355, 356, 449, 465, 611	Hesperetin-7-*O*-neohesperidoside
8	5.34		595	270, 475, 481, 529, 595	Apigenin-6-*C*-glucoside-7-*O*-glucoside
9	5.44		355	145, 163, 355	Chlorogenic acid
10	5.55		291	119, 123, 139, 147, 151, 165, 207, 263, 261	Epicatechin
11	9.36		655	331, 333.2. 493, 494	Malvidin-3,5-di-*O*-glucoside chloride
12	5.88		593	107, 163, 167, 189, 255, 265, 283, 287, 301, 342, 430, 446, 463	Acacetin-7-*O*-rutinoside
13	6.10		579	112, 579	Procyanidin B2
14	6.11		449	199, 299, 300, 310, 323, 325, 329, 337, 339, 349, 353, 377, 383, 395,431, 449	Luteolin-6-*C*-glucoside
15	6.23	609		293, 401, 454, 465, 563, 577, 607, 609	Luteolin-3′,7-di-*O*-glucoside
16	6.28		597	303,465	Quercetin-3-*O*-arabinoglucoside
17	6.39	591		283, 429	Acacetin-7-*O*-rutinoside isomer
18	6.50	625		301, 463, 625	Quercetin- 3,4′-*O*-di-glucopyranoside
19	6.54	463		218, 271, 300, 301, 394, 463	Delphinidin-3-*O*-glucopyranoside
20	6.58		593	285, 431	Acacetin-7-*O*-neohesperidoside
21	6.72		611	303, 465, 611	Rutin
22	6.73		451	153, 163, 179, 289	Eriodictyol-7-*O*-glucoside
23	6.78		451	107, 149, 153, 167, 195, 215, 243, 271, 289	Okanin-4′-*O*-glucoside
24	6.83	623		161, 315, 461, 623	Isorhamnetin-3-*O*-rutinoside
25	6.85		433	165, 271, 283, 284, 295, 297, 309, 313, 323, 337, 343, 349, 351, 361, 367, 379, 415, 433	Apigenin-8-*C*-glucoside
26	6.89		581	287, 449	Cyanidin -3-*O*-(2‴-*O*-xylopyranosyl-beta glucopyranoside)
27	6.99	463		301,	Isoquercitrin
28	7.06		611	303	Hyperoside
29	7.10		341	179	Esculin
30	7.13		451	107, 149, 150, 153, 167, 195, 215, 243, 271, 288, 289	Isookanin-7-glucoside
31	7.23	477		209, 227, 364, 431, 433, 477	Isorhamnetin-3-*O*-glucoside
32	7.26		417	417	Daidzin-8-*C*-glucoside
33	7.33	447		285, 299, 300, 301, 447	Quercetin-7-*O*-rhamnoside
34	7.33		595	287, 449, 595	Kaempferol-3-*O*-rutinoside
35	7.41		417	255, 398, 417	4′-hydroxyisoflavone-7-glucoside
36	7.49		449	287	Cyanidin-3-glucoside
37	7.68		135	79, 135	Cinnamyl alcohol
38	7.74		133	51, 53, 57, 59, 70, 72, 73, 75, 77, 79, 91, 103, 105, 115, 116, 117, 118, 131, 133	Cinnamaldehyde
39	7.78		449	71, 85, 287, 303	Quercitrin
40	7.78	611		285, 565, 611	Neohesperedin dihydrochalcone
41	7.87		433	266, 271, 433	Apigenin-7-*O*-glucoside
42	7.89		435	81, 227, 255, 273, 303, 435	Naringenin-7-*O*-glucoside
43	8.32	595		265, 269, 594.6, 595	Eriodictyol-7-*O*-neohesperidoside
44	8.43		479	303	Quercetin-3-*O*-gluccouronide
45	8.53		433	71, 85, 287	Afzelin
46	8.75	461		183, 208, 223, 225, 237, 324, 331, 392, 443, 461	Kaempferol-3-*O*-glucouroide
47	8.94		463	107, 163, 167, 189, 255, 265, 283, 287, 301, 342, 430, 446, 463	Peonidine-3-*O*-glucoside chloride
48	9.17		431	237, 257, 269	Ononin
49	9.20	593		284, 285, 385, 547, 593	Kaempferol-3-*O*-(6-*p*-coumaryl)-glucoside
50	9.75		303	149, 153, 229, 257, 285, 303	Quercetin
51	10.88		303	153, 177, 303	Hesperetin
52	10.40	286.9	289	153, 163, 289	3′,4′,5,7-tetrahydroxy flavanone (Eriodictyol)
53	10.44	445		164, 195, 207, 235, 237, 445	Baicalen-7-*O*-glucuronide
54	10.76		285	133, 153, 242, 270, 285	Acacetin
55	11.16		271	119, 153, 271	Apigenin
56	11.43		287	121, 135, 149, 153, 157, 184, 203, 213, 231, 259, 287	Luteolin
57	11.53		305	70, 112, 116, 153, 179, 200, 244, 270, 287, 305	Taxifolin
58	11.62	449		287, 387, 449	Eriodictyol-7-*O*-glucoside
59	11.76	299		119, 135, 179, 253, 298, 299	Kaempferide
60	11.94		181	51, 68, 83, 91, 103, 121, 138, 149, 163, 181	Caffeic acid
61	12.37		271	153, 243, 253, 271	Genistein
62	12.60		179	67, 91, 95, 105, 115, 123, 131, 133, 147, 163, 179	Daphnetin
63	12.63		269	115, 136, 137, 149, 181, 191, 209, 213, 223, 225, 237, 257, 269	Formononetin
64	12.89		273	81, 147, 153, 227, 273	Naringenin
65	12.90		255	81, 137, 199, 227, 255	Daidzein
66	13.03		219	176, 219	4-Methylumbelliferyl acetate
67	13.09	153		65, 108, 109, 112, 153	3,4-Dihydroxybenzoic acid
68	13.23		449	291, 449	Luteolin-8-*C*-glucoside
69	14.16		285	84, 268, 285	Acacetin isomer
70	14.58		219	77	4-Methylumbelliferone
71	15.06		289	153, 163	Aromadendrin
72	16.27		271	113, 169, 271	Baicalen
73	16.73		493	331	Malvidin-3-*O*-galactoside
74	17.48		317	302, 303	3-*O*-methyl quercetin
75	17.49		317	149, 167, 317	Rhamnetin
76	17.55		317	153, 163, 317	Isorhamnetin
77	17.71		287	153, 161	Isosakuranetin
78	20.15		273	85, 131, 273	16-Hydroxyhexadecanoic acid
79	22.59		209	79, 107, 135, 148, 163, 191, 209	3,4-dimethoxy cinnamic acid
80	23.14		299	299	Methyl octadecanoate
81	23.42		281	119, 281	Linoleic acid

**Table 2:** Mechanism of the antiviral activity of APEE against SARS-CoV-2.

Mode of Action	Conc.* (mg/mL)	Virus Control (PFU/mL)	Viral Titer Post-Treatment (PFU/mL)	Viral Inhibition (%)
Virucidal	0.25	4.5 × 10^5^	0.2 × 10^5^	95.6%
	0.125		0.4 × 10^5^	91.1%
	0.0625		0.4 × 10^5^	91.1%
	0.0312		0.5 × 10^5^	88.9%
Replication	0.25	1.0 × 10^5^	0.75 × 10^5^	25%
	0.125		0.85 × 10^5^	15%
	0.0625		0.9 × 10^5^	10%
	0.0312		1.0 × 10^5^	0%
Adsorption	0.25	1.33 × 10^5^	0.25 × 10^5^	81.2%
	0.125		0.45 × 10^5^	66.2%
	0.0625		0.69 × 10^5^	48.1%
	0.0312		1.03 × 10^5^	22.6%

* The APEE antiviral mechanisms of action against SARS-CoV-2 were investigated at concentrations relatively higher than IC_50_ for improved comprehension of the mechanisms of action.

**Table 3:** Effects of APEE Pre-treatment on lung W/D ratio, lung NO, Serum TAC, and MPO activity in LPS-induced acute lung injury in mice.

	Lung W/D Ratio	Lung NO Content (nmol/g Tissue)	Serum TAC (Mm/L)	Lung MPO Activity (µM/min/g Tissue)
Control saline	7.6 ± 1.1	12.6 ± 0.85	1.92 ± 0.12	2.81 ± 0.29
LPS	11.5 ± 0.85 ^a^	23.6 ± 1.6 ^a^	0.53 ± 0.085 ^a^	11.36 ± 0.77 ^a^
APEE 200	9.5 ± 0.94 ^b^	17.8 ± 1.3 ^b^	0.9 ± 0.06 ^b^	6.8 ± 0.84 ^b^
APEE 250	7.8 ± 1.01 ^bc^	13.1 ± 1.1 ^bc^	1.89 ± 0.15 ^bcd^	3.01 ± 0.29 ^bcd^
APEE 250	8.5 ± 0.81 ^b^	15 ± 1.5 ^b^	1.5 ± 0.18 ^b^	3.85 ± 0.36 ^b^

Acute lung injury was urged by I.P. injection of LPS (10 mg/kg). APEE 200, 250, and 300 were given I.P. 30 min before LPS injection. Results were expressed as mean ± SD (*n* = 10/group). The experiments were performed in three independent replicates. Significant difference vs. ^a^ respective control, ^b^ respective LPS group, ^c^ respective APEE 200 group, ^d^ respective APEE 300 group each at *p* < 0.05.

## Data Availability

Data is contained within article and Supplementary Materials.

## Supplementary Materials

The following are available online at https://www.mdpi.com/article/10.3390/ph14121313/s1, Figure S1. The total ion chromatogram (TIC) of APEE (A) negative and (B) positive mode. Figure S2. DPPH Assay Figure S3. ABTS Assay Figure S4. FRAP Assay. Figure S5. Antioxidant effect of Trolox on the decay of fluorescein in ORAC assay. Figure S6. Signal curve of Sample and blank indicating the decay of fluoresceine upon applying the sample. Table S1: Sequences of the utilized primers (in vitro). Table S2. Primers used and their sequence (in vivo).

## References

[1] Zhou P., Yang X.-L., Wang X.-G., Hu B., Zhang L., Zhang W., Si H.-R., Zhu Y., Li B., Huang C.-L. (2020). A pneumonia outbreak associated with a new coronavirus of probable bat origin. Nature.

[2] Huang C., Wang Y., Li X., Ren L., Zhao J., Hu Y., Zhang L., Fan G., Xu J., Gu X. (2020). Clinical features of patients infected with 2019 novel coronavirus in Wuhan, China. Lancet.

[3] Boban M. (2021). Novel coronavirus disease (COVID-19) update on epidemiology, pathogenicity, clinical course and treatments. Int. J. Clin. Pract..

[4] Aguilar R.B., Hardigan P., Mayi B., Sider D., Piotrkowski J., Mehta J.P., Dev J., Seijo Y., Camargo A.L., Andux L. (2020). Current understanding of COVID-19 clinical course and investigational treatments. Front. Med..

[5] Lin S.-N., Rui J., Chen Q.-P., Zhao B., Yu S.-S., Li Z.-Y., Zhao Z.-Y., Wang Y., Zhu Y.-Z., Xu J.-W. (2021). Effectiveness of potential antiviral treatments in COVID-19 transmission control: A modelling study. Infect. Dis. Poverty.

[6] WHO (2021). WHO Coronavirus (COVID-19) Dashboard (26-4-2021).

[7] Duman N., ALzaidi Z., Aynekin B., Taskin D., Demirors B., Yildirim A., Sahin I.O., Bilgili F., Turanli E.T., Beccari T. (2021). COVID-19 vaccine candidates and vaccine development platforms available worldwide. J. Pharm. Anal..

[8] Sytar O., Brestic M., Hajihashemi S., Skalicky M., Kubeš J., Lamilla-Tamayo L., Ibrahimova U., Ibadullayeva S., Landi M. (2021). COVID-19 prophylaxis efforts based on natural antiviral plant extracts and their compounds. Molecules.

[9] Huang J., Tao G., Liu J., Cai J., Huang Z., Chen J.-X. (2020). Current prevention of COVID-19: Natural products and herbal medicine. Front. Pharmacol..

[10] Kim J.-J., Jiang J., Shim D.-W., Kwon S.-C., Kim T.-J., Ye S.-K., Kim M.-K., Shin Y.-K., Koppula S., Kang T.-B. (2012). Anti-inflammatory and anti-allergic effects of *Agrimonia pilosa* Ledeb extract on murine cell lines and OVA-induced airway inflammation. J. Ethnopharmacol..

[11] Kim S.B., Hwang S.H., Suh H.-W., Lim S.S. (2017). Phytochemical analysis of *Agrimonia pilosa* Ledeb, its antioxidant activity and aldose reductase inhibitory potential. Int. J. Mol. Sci..

[12] Zhu L., Tan J., Wang B., He R., Liu Y., Zheng C. (2009). Antioxidant activities of aqueous extract from *Agrimonia pilosa* Ledeb and its fractions. Chem. Biodivers..

[13] Kato H., Li W., Koike M., Wang Y., Koike K. (2010). Phenolic glycosides from *Agrimonia pilosa*. Phytochemistry.

[14] Pang H., Zhu Y., Qiao P., Wen D.-Z. (2006). Genetic toxicity of *Agrimonia pilosa* Ledeb in male mouse genital cells. J. Jilin Univ. (Med. Ed.).

[15] Park J.-H., Ra J.-S., Kwon J.E., Her Y.-M., Choe T.H., Lee Y.-S., Suh H.J., Shin S.-Y., Park D.W., Kwak H.-H. (2020). Evaluation of genetic toxicity, acute and sub-chronic oral toxicity and systemic safety of *Agrimonia pilosa* and *Rhus gall* 50% ethanolic extract mixture (APRG64) in vitro and in vivo (rodent and non-rodent animal models). Toxicol. Res..

[16] Shin W.J., Lee K.H., Park M.H., Seong B.L. (2010). Broad-spectrum antiviral effect of *Agrimonia pilosa* extract on influenza viruses. Microbiol. Immunol..

[17] Tsimogiannis D., Samiotaki M., Panayotou G., Oreopoulou V. (2007). Characterization of flavonoid subgroups and hydroxy substitution by HPLC-MS/MS. Molecules.

[18] Kachlicki P., Piasecka A., Stobiecki M., Marczak Ł. (2016). Structural characterization of flavonoid glycoconjugates and their derivatives with mass spectrometric techniques. Molecules.

[19] Nakata R., Yoshinaga N., Teraishi M., Okumoto Y., Huffaker A., Schmelz E.A., Mori N. (2018). A fragmentation study of isoflavones by IT-TOF-MS using biosynthesized isotopes. Biosci. Biotechnol. Biochem..

[20] Flamini R. (2013). Recent applications of mass spectrometry in the study of grape and wine polyphenols. Int. Sch. Res. Not..

[21] Sun C., Wang Y., Sun S., Chen X., Shi X., Fang H., Zhang Y., Fang Z. (2020). Fragmentation pathways of protonated coumarin by ESI-QE-Orbitrap-MS/MS coupled with DFT calculations. J. Mass Spectrom..

[22] Tine Y., Renucci F., Costa J., Wélé A., Paolini J. (2017). A method for LC-MS/MS profiling of coumarins in *Zanthoxylum zanthoxyloides* (Lam.) B. Zepernich and Timler extracts and essential oils. Molecules.

[23] Šuković D., Knežević B., Gašić U., Sredojević M., Ćirić I., Todić S., Mutić J., Tešić Ž. (2020). Phenolic profiles of leaves, grapes and wine of grapevine variety vranac (*Vitis vinifera* L.) from Montenegro. Foods.

[24] Remali J., Aizat W.M. (2020). A review on plant bioactive compounds and their modes of action against coronavirus infection. Front. Pharmacol..

[25] Chen L., Kang Y.-H. (2014). Antioxidant activities of *Agrimonia pilosa* ledeb: In vitro comparative activities of its different fractions. Korean J. Plant Resour..

[26] Mostafa A., Kandeil A., Elshaier A.M.M.Y., Kutkat O., Moatasim Y., Rashad A.A., Shehata M., Gomaa M.R., Mahrous N., Mahmoud S.H. (2020). FDA-approved drugs with potent in vitro antiviral activity against severe acute respiratory syndrome coronavirus 2. Pharmaceuticals.

[27] Nie C., Trimpert J., Moon S., Haag R., Gilmore K., Kaufer B.B., Seeberger P.H. (2021). In vitro efficacy of Artemisia extracts against SARS-CoV-2. bioRxiv.

[28] Saito K., Fukasawa M., Shirasago Y., Suzuki R., Osada N., Yamaji T., Wakita T., Konishi E., Hanada K. (2020). Comparative characterization of Flavivirus production in two cell lines: Human hepatoma-derived Huh7. 5.1–8 and african green monkey kidney-derived vero. PLoS ONE.

[29] Roshdy W.H., Rashed H.A., Kandeil A., Mostafa A., Moatasim Y., Kutkat O., Abo Shama N.M., Gomaa M.R., El-Sayed I.H., El Guindy N.M. (2020). EGYVIR: An immunomodulatory herbal extract with potent antiviral activity against SARS-CoV-2. PLoS ONE.

[30] Zannella C., Giugliano R., Chianese A., Buonocore C., Vitale G.A., Sanna G., Sarno F., Manzin A., Nebbioso A., Termolino P. (2021). Antiviral Activity of *Vitis vinifera* Leaf Extract against SARS-CoV-2 and HSV-1. Viruses.

[31] Plante K.S., Dwivedi V., Plante J.A., Fernandez D., Mirchandani D., Bopp N., Aguilar P.V., Park J.-G., Tamayo P.P., Delgado J. (2021). Antiviral activity of oleandrin and a defined extract of *Nerium oleander* against SARS-CoV-2. Biomed. Pharmacother..

[32] Jo S., Kim S., Shin D.H., Kim M.-S. (2020). Inhibition of SARS-CoV 3CL protease by flavonoids. J. Enzym. Inhib. Med. Chem..

[33] Viola A., Munari F., Sánchez-Rodríguez R., Scolaro T., Castegna A. (2019). The metabolic signature of macrophage responses. Front. Immunol..

[34] Dong J., Li J., Cui L., Wang Y., Lin J., Qu Y., Wang H. (2018). Cortisol modulates inflammatory responses in LPS-stimulated RAW264. 7 cells via the NF-κB and MAPK pathways. BMC Vet. Res..

[35] Sharifi-Rad M., Anil Kumar N.V., Zucca P., Varoni E.M., Dini L., Panzarini E., Rajkovic J., Tsouh Fokou P.V., Azzini E., Peluso I. (2020). Lifestyle, oxidative stress, and antioxidants: Back and forth in the pathophysiology of chronic diseases. Front. Physiol..

[36] Matthay M.A., Ware L.B., Zimmerman G.A. (2012). The acute respiratory distress syndrome. J. Clin. Investig..

[37] An X., Sun X., Hou Y., Yang X., Chen H., Zhang P., Wu J. (2019). Protective effect of oxytocin on LPS-induced acute lung injury in mice. Sci. Rep..

[38] Imai Y., Kuba K., Neely G.G., Yaghubian-Malhami R., Perkmann T., van Loo G., Ermolaeva M., Veldhuizen R., Leung Y.C., Wang H. (2008). Identification of oxidative stress and Toll-like receptor 4 signaling as a key pathway of acute lung injury. Cell.

[39] Le Q.U., Joshi R.K., Lay H.L., Chang M. (2018). *Agrimonia pilosa* Ledeb: Phytochemistry, Ethnopharmacology, Pharmacology of an important traditional herbal medicine. J. Pharmacogn. Phytochem..

[40] Kim C.Y., Yu Q.-M., Kong H.-J., Lee J.-Y., Yang K.-M., Seo J.-S. (2020). Antioxidant and anti-inflammatory activities of *Agrimonia pilosa* Ledeb. extract. Evid.-Based Complement. Altern. Med..

[41] Li C., Wang M., Sui J., Zhou Y., Chen W. (2021). Protective mechanisms of *Agrimonia pilosa* Ledeb in dextran sodium sulfate-induced colitis as determined by a network pharmacology approach. Acta Biochim. Biophys. Sin..

[42] Kim D.-S., Park K.-E., Kwak Y.-J., Bae M.-K., Bae S.-K., Jang I.-S., Jang H.-O. (2020). *Agrimonia pilosa* Ledeb Root Extract: Anti-Inflammatory Activities of the Medicinal Herb in LPS-Induced Inflammation. Am. J. Chin. Med..

[43] Feng J.-H., Kim H.-Y., Sim S.-M., Zuo G.-L., Jung J.-S., Hwang S.-H., Kwak Y.-G., Kim M.-J., Jo J.-H., Kim S.-C. (2021). The Anti-Inflammatory and the Antinociceptive Effects of Mixed *Agrimonia pilosa* Ledeb. and *Salvia miltiorrhiza* Bunge Extract. Plants.

[44] Jang H.H., Nam S.Y., Kim M.J., Kim J.B., Choi J.S., Kim H.R., Lee Y.M. (2017). *Agrimonia pilosa* Ledeb. aqueous extract improves impaired glucose tolerance in high-fat diet-fed rats by decreasing the inflammatory response. BMC Complement. Altern. Med..

[45] Cecchini R., Cecchini A.L. (2020). SARS-CoV-2 infection pathogenesis is related to oxidative stress as a response to aggression. Med. Hypotheses.

[46] Karkhanei B., Ghane E.T., Mehri F. (2021). Evaluation of oxidative stress level: Total antioxidant capacity, total oxidant status and glutathione activity in patients with Covid-19. New Microbes New Infect..

[47] Delgado-Roche L., Mesta F. (2020). Oxidative stress as key player in severe acute respiratory syndrome coronavirus (SARS-CoV) infection. Arch. Med Res..

[48] Kawasaki M., Kuwano K., Hagimoto N., Matsuba T., Kunitake R., Tanaka T., Maeyama T., Hara N. (2000). Protection from lethal apoptosis in lipopolysaccharide-induced acute lung injury in mice by a caspase inhibitor. Am. J. Pathol..

[49] Huang W., Jin S., Yang W., Tian S., Meng C., Deng H., Wang H. (2020). Protective effect of *Agrimonia pilosa* polysaccharides on dexamethasone-treated MC3T3-E1 cells via Wnt/β-Catenin pathway. J. Cell. Mol. Med..

[50] Lee Y.-G., Kang K.W., Hong W., Kim Y.H., Oh J.T., Park D.W., Ko M., Bai Y.-F., Seo Y.-J., Lee S.-M. (2021). Potent antiviral activity of *Agrimonia pilosa*, *Galla rhois*, and their components against SARS-CoV-2. Bioorg. Med. Chem..

[51] Attallah N.G.M., Negm W.A., Elekhnawy E., Elmongy E.I., Altwaijry N., El-Haroun H., El-Masry T.A., El-Sherbeni S.A. (2021). Elucidation of Phytochemical Content of Cupressus macrocarpa Leaves: In Vitro and In Vivo Antibacterial Effect against Methicillin-Resistant *Staphylococcus aureus* Clinical Isolates. Antibiotics.

[52] Attard E. (2013). A rapid microtitre plate Folin-Ciocalteu method for the assessment of polyphenols. Open Life Sci..

[53] Kiranmai M., Kumar C.M., Mohammed I. (2011). Comparison of total flavanoid content of *Azadirachta indica* root bark extracts prepared by different methods of extraction. Res. J. Pharm. Biol. Chem. Sci..

[54] Boly R., Lamkami T., Lompo M., Dubois J., Guissou I. (2016). DPPH free radical scavenging activity of two extracts from *Agelanthus dodoneifolius* (Loranthaceae) leaves. Int. J. Toxicol. Pharmacol. Res..

[55] Chen Z., Bertin R., Froldi G. (2013). EC50 estimation of antioxidant activity in DPPH assay using several statistical programs. Food Chem..

[56] Arnao M.B., Cano A., Acosta M. (2001). The hydrophilic and lipophilic contribution to total antioxidant activity. Food Chem..

[57] Benzie I.F., Strain J.J. (1996). The ferric reducing ability of plasma (FRAP) as a measure of “antioxidant power”: The FRAP assay. Anal. Biochem..

[58] Liang Z., Cheng L., Zhong G.-Y., Liu R.H. (2014). Antioxidant and antiproliferative activities of twenty-four *Vitis vinifera* grapes. PLoS ONE.

[59] Payne S. (2017). Methods to study viruses. Viruses.

[60] Zhang J., Zhan B., Yao X., Gao Y., Shong J. (1995). Antiviral activity of tannin from the pericarp of *Punica granatum* L. against genital Herpes virus in vitro. Zhongguo Zhong Yao Ya Zhi Zhongguo Zhongyao Zazhi China J. Chin. Mater. Med..

[61] Kuo Y.-C., Lin L.-C., Tsai W.-J., Chou C.-J., Kung S.-H., Ho Y.-H. (2002). Samarangenin B from Limonium sinense suppresses herpes simplex virus type 1 replication in Vero cells by regulation of viral macromolecular synthesis. Antimicrob. Agents Chemother..

[62] Schuhmacher A., Reichling J., Schnitzler P. (2003). Virucidal effect of peppermint oil on the enveloped viruses herpes simplex virus type 1 and type 2 in vitro. Phytomedicine.

[63] Chan-Zapata I., Canul-Canche J., Fernández-Martín K., Martín-Quintal Z., Torres-Romero J.C., Lara-Riegos J.C., Ramírez-Camacho M.A., Arana-Argáez V.E. (2018). Immunomodulatory effects of the methanolic extract from *Pouteria campechiana* leaves in macrophage functions. Food Agric. Immunol..

[64] Ezzat M.I., Hassan M., Abdelhalim M.A., El-Desoky A.M., Mohamed S.O., Ezzat S.M. (2021). Immunomodulatory effect of Noni fruit and its isolates: Insights into cell-mediated immune response and inhibition of LPS-induced THP-1 macrophage inflammation. Food Funct..

[65] Nho J.H., Jang J.H., Lee H.J., Yang B., Woo K.W., Kim A.H., Seo J.W., Hwang T.Y., Cho H.W., Jung H.K. (2019). Preventive effect of the water extract of *Agrimonia pilosa* Ledeb and micronucleus assay-based evaluation of genotoxicity in gastritis animal models. Korean J. Med. Crop Sci..

[66] Park S.-H., Sim Y.-B., Kang Y.-J., Lee J.-K., Lim S.-S., Suh H.-W. (2012). Effect of *Agrimonia pilosa* Ledeb extract on the antinociception and mechanisms in mouse. Korean J. Physiol. Pharmacol..

[67] Koracevic D., Koracevic G., Djordjevic V., Andrejevic S., Cosic V. (2001). Method for the measurement of antioxidant activity in human fluids. J. Clin. Pathol..

[68] El-Mahdy N.A., El-Sayad M.E.S., El-Kadem A.H., Abu-Risha S.E.S. (2021). Targeting IL-10, ZO-1 gene expression and IL-6/STAT-3 trans-signaling by a combination of atorvastatin and mesalazine to enhance anti-inflammatory effects and attenuates progression of oxazolone-induced colitis. Fundam. Clin. Pharmacol..

[69] Livak K.J., Schmittgen T.D. (2001). Analysis of relative gene expression data using real-time quantitative PCR and the 2− ΔΔCT method. Methods.

[70] Pound J.D. (1998). Immunochemical Protocols.

[71] Lillehoj E.P., Malik S.V. (1994). Antibody Techniques.

