# Supplementary material for: Promising Antiviral Activity of Agrimonia pilosa Phytochemicals against Severe Acute Respiratory Syndrome Coronavirus 2 Supported with In Vivo Mice Study
Source: Pharmaceuticals (Basel). 2021 Dec 16;14(12):1313. doi: 10.3390/ph14121313 (PMC8709118; doi:10.3390/ph14121313)
Supplement: Supplementary file 1 [file pharmaceuticals-14-01313-s001.zip › pharmaceuticals-1469881-supplementary.pdf]

Table S1. Sequences of the utilized primers (in vitro).

Gene	Primer sequence*
COX-2	F 5′-TTCAAATGAGATTGTGGGAAAATTGCT-3′<br>R 5′-AGATCATCTCTGCCTGAGTATCTT-3′
iNOS	F 5′-TCTTGGTCAAAGCTGTGCTC-3′<br>R 5′-CATTGCCAAACGTACTGGTC-3′
IL-6	F 5′-AAAGAGGCACTGGCAGAAAA-3′<br>R 5′-AGCTCTGGCTTGTTCCTCAC-3′
TNF-α	F 5'-ATGAGCACTGAAAGCATGATC-3'<br>R 5'-TCACAGGGCAATGATCCCAAAGTAGACCTGCCC-3'
NF-κB	F 5′-GCGGGAGAGGGGATTCCCTGCGGCCCCG-3′<br>R 5′-CGGGGCCGCAGGGAATCCCCTCTCCCGC-3′
GAPDH	F 5'-ACCACAGTCCATGCCATCAC-3'<br>R 5'-TCCACCACCCTGTTGCTGTA-3'

* F stands for forward and R stands for reverse.

Table S2. Primers used and their sequence (in vivo).

Target gene	Probe
**IL-18**	TAA GGA TAC GGA CTA CGG CT<br>GTT GGT GGA GGT CTG AGT TTA
**IL-10**	CCAAGCCTTATCGGAAATGA<br>TTTTCACAGGGGAGAAATCG
**HO-1**	5′-CGACAGCATGTCCCAGGATT-3′<br>5′-TCGCTCTATCTCCTCTTCCAGG-3′
**Caspase-1**	AACACCCACTCGTACACGTC<br>TGAGGTCAACATCAGCTCCG
**Caspase-3**	5'-TGAAGGCAAGGT GCTAAA-3'<br>5'-CTGGCTCAAACCACATTCTC-3'
**β-actin**	CACGATGGAGGGGCCGGACTCATC<br>TAAAGACCTCTATGCCAACACAGT

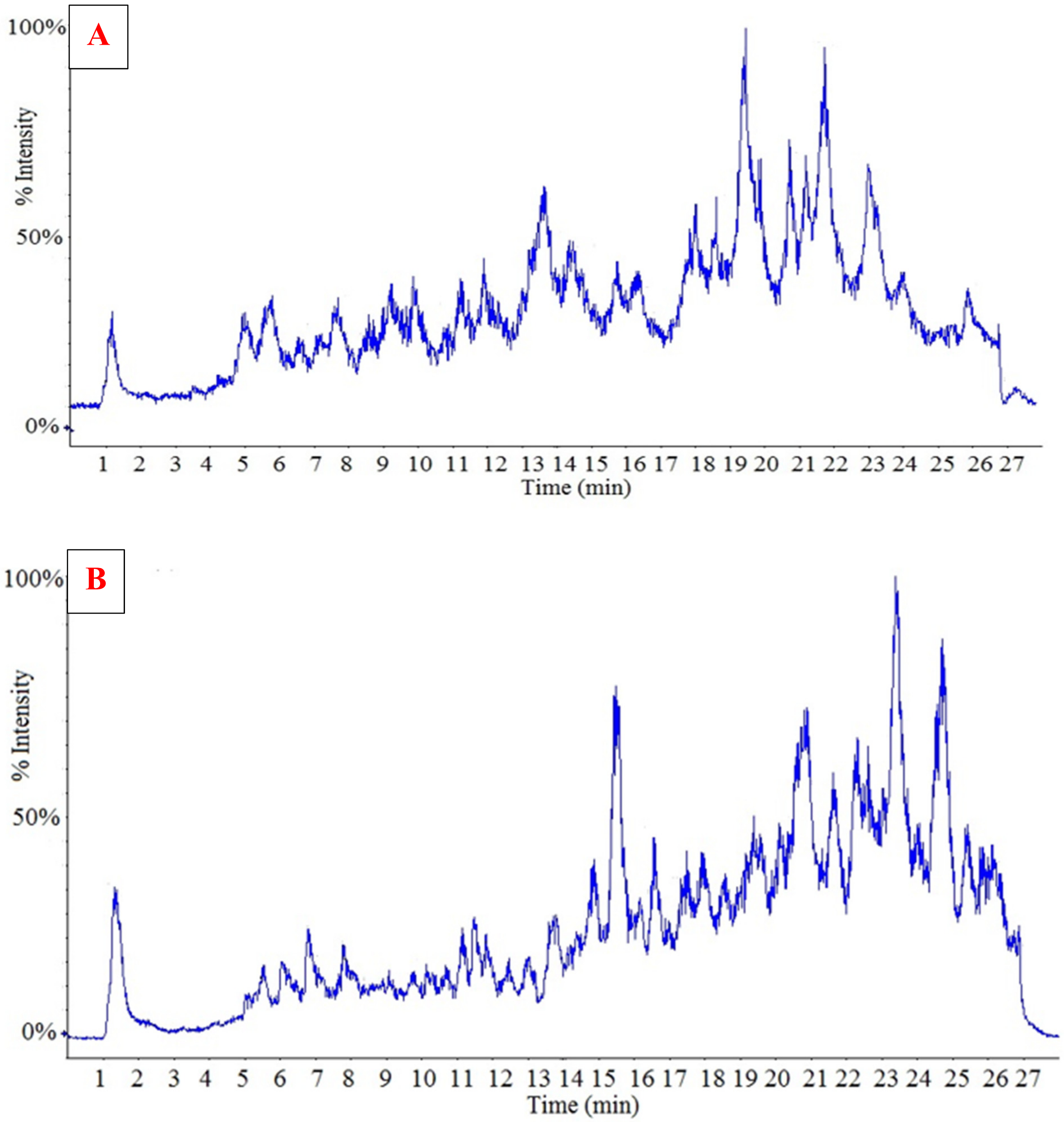

Figure S1. The total ion chromatogram (TIC) of APEE (A) negative and (B) positive modes.

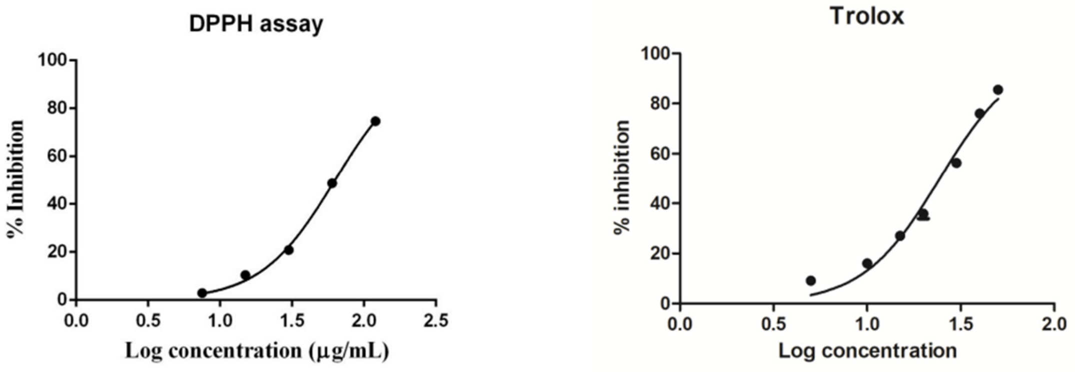

Figure S2. DPPH Assay.

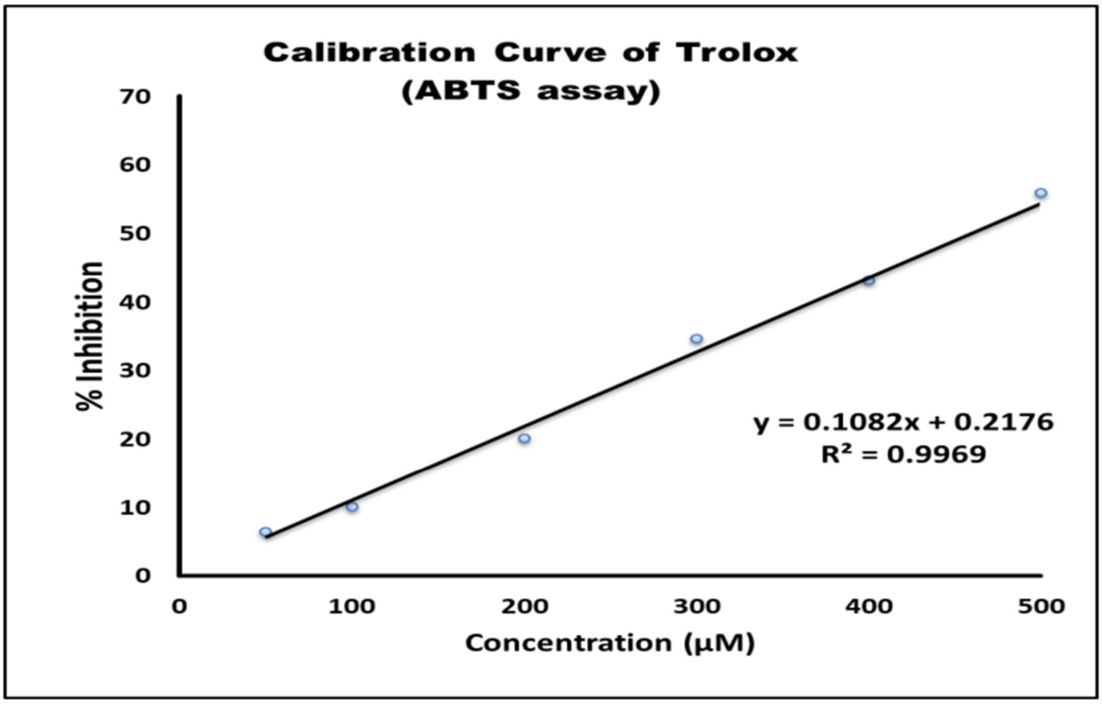

Figure S3. ABTS Assay. The average of the readings of the 6 replicates was taken after subtraction from the Blank (ABTS reagent+ solvent).

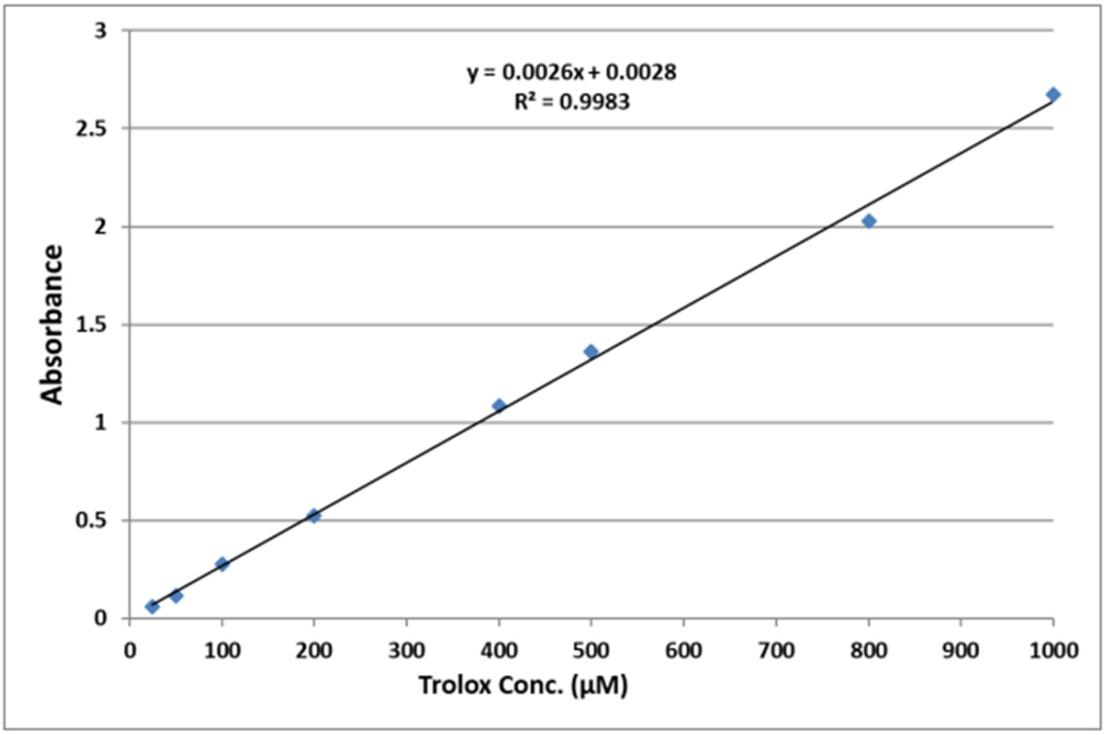

Figure S4. FRAP Assay. The average of the readings of the 6 replicates was taken after subtraction from the Blank (FRAP reagent+ solvent).

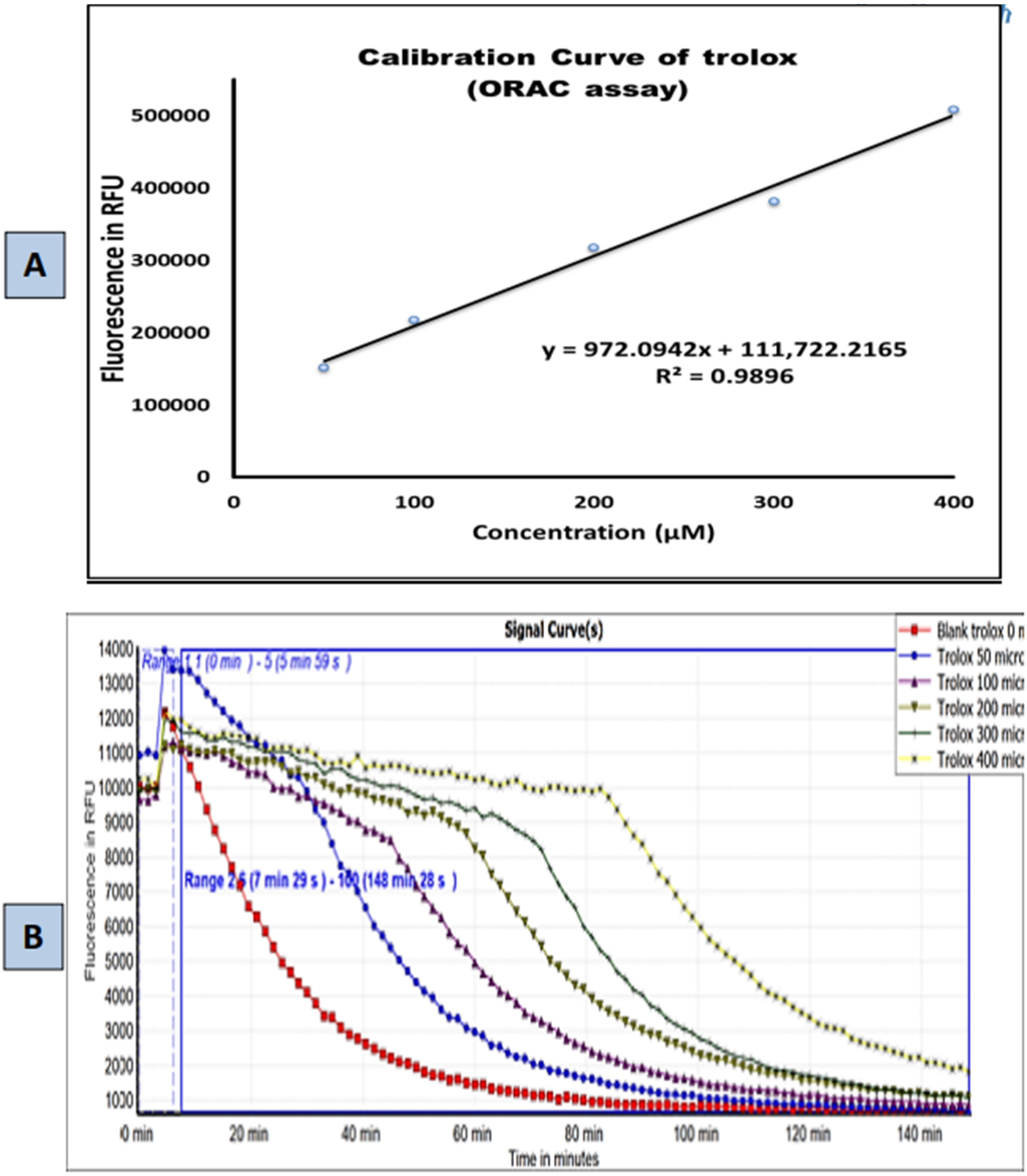

Figure S5. Antioxidant effect of Trolox on the decay of fluorescein in ORAC assay. (A) blank corrected linear regression curve of Trolox. (B) signal curves of different Trolox concentrations and balnk indicating the decay of fluoresceine with different concentrations of Trolox.

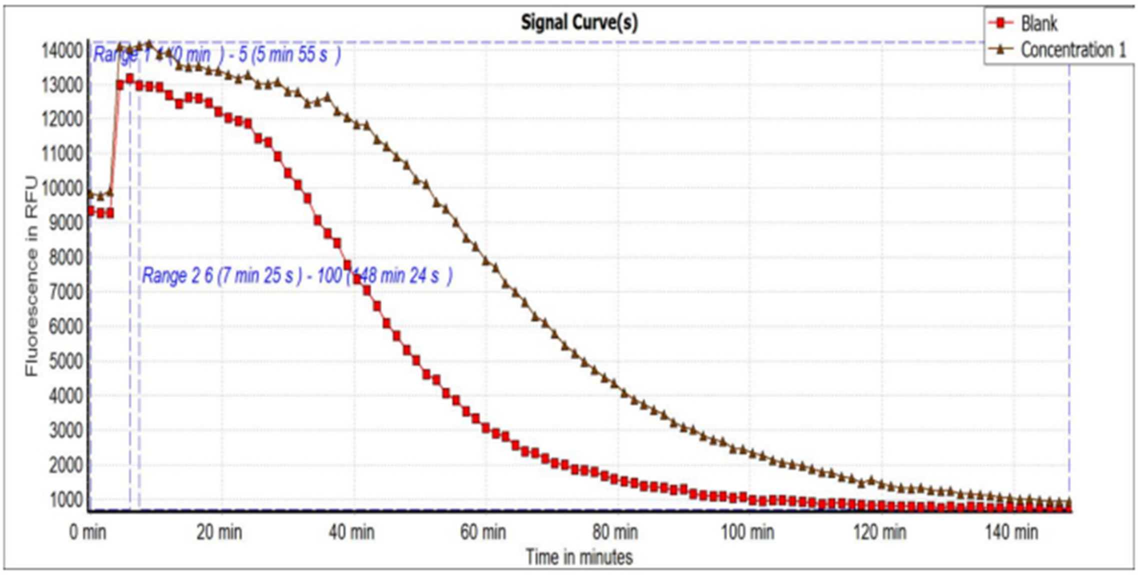

Figure S6. Signal curve of Sample and blank indicating the decay of fluoresceine upon applying the sample.
